# Cost-Effectiveness of Tepotinib Versus Capmatinib for the Treatment of Adult Patients With Metastatic Non–Small Cell Lung Cancer Harboring Mesenchymal–Epithelial Transition Exon 14 Skipping

**DOI:** 10.1016/j.jval.2022.11.018

**Published:** 2022-12-08

**Authors:** Mo Yang, Helene Vioix, Rameet Sachdev, Matthew Stargardter, Jon Tosh, Boris M. Pfeiffer, Paul K. Paik

**Affiliations:** EMD Serono, Rockland, MA, USA; healthcare business of Merck KGaA, Darmstadt, Germany; Evidera, Bethesda MD, USA; Evidera, Bethesda MD, USA; Evidera, London, UK; healthcare business of Merck KGaA, Darmstadt, Germany; Memorial Sloan Kettering Cancer Center, New York City, NY, USA

**Keywords:** cost-effectiveness, mesenchymal–epithelial transition factor gene exon 14 skipping, metastatic non–small cell lung cancer, tepotinib, tyrosine kinase inhibitors, VISION trial

## Abstract

**Objectives:**

From the US Medicare perspective, this study compared the cost-effectiveness of tepotinib and capmatinib for treating metastatic non–small cell lung cancer with tumors harboring mesenchymal–epithelial transition factor gene exon 14 skipping.

**Methods:**

A 3-state partitioned survival model assessed outcomes over a lifetime horizon. Parametric survival analysis of the phase 2 VISION trial informed clinical inputs for tepotinib. Capmatinib inputs were captured using hazard ratios derived from an unanchored matching-adjusted indirect comparison study and published literature. National cost databases, trial data, and literature furnished drug, treatment monitoring, and disease/adverse event management expenditures (2021 US dollars) and utility inputs. Outcomes were discounted at 3% annually.

**Results:**

In the base case, tepotinib dominated capmatinib in frontline settings (incremental discounted quality-adjusted life-years [QALYs] and costs of 0.2127 and −$47 756, respectively) while realizing an incremental cost-effectiveness ratio of $274 514/QALY in subsequent lines (incremental QALYs and costs of 0.3330 and $91401, respectively). In a line agnostic context, tepotinib produced an incremental cost-effectiveness ratio of $105 383/QALY (incremental QALYs and costs of 0.2794 and $29 447, respectively). Sensitivity and scenarios analyses for individual lines typically supported the base case, whereas those for the line agnostic setting suggested sensitivity to drug acquisition costs and efficacy inputs.

**Conclusions:**

Tepotinib could be cost-effective versus capmatinib in frontline and line agnostic contexts, considering the range of willingness-to-pay thresholds recommended by the Institute for Clinical and Economic Review ($100 000-$150 000/QALY). Tepotinib could be cost-effective in subsequent lines at higher willingness-to-pay levels. These results are to be interpreted cautiously, considering uncertainty in key model inputs.

## Introduction

Lung cancer is a leading cause of cancer-related mortality in the United States, accounting for an estimated 21.7% of all cancer deaths in the United States in 2021.^1^ Non–small cell lung cancer (NSCLC) accounts for approximately 80% to 85% of cases.^2^ Despite innovative therapies, NSCLC survival rates remain low, with the 5-year survival rate in patients diagnosed of distant metastatic (stage IV) cancer being approximately 6.3% in the United States.^1^

Approximately 3% to 4% of NSCLCs harbor mesenchymal–epithelial transition factor gene exon 14 (*MET*ex14) skipping, which has been recognized as an oncogenic driver.^3-7^ Patients with NSCLC harboring *MET*ex14 skipping tend to be older,^8^ to exhibit a nonsquamous histology,^9,10^ and to have poorer prognoses because of higher rates of brain, bone, and liver metastases.^11-13^ Until tyrosine kinase inhibitors (TKIs) were approved, there were no therapies approved in the United States to specifically treat patients with NSCLC harboring *MET*ex14 skipping, generally leaving this patient subgroup with recourse only to approved nontargeted therapies.

In May 2020, capmatinib was the first TKI approved by the US Food and Drug Administration (FDA) for adult patients with metastatic NSCLC (mNSCLC) whose tumors have a mutation that leads to *MET*ex14 skipping, as detected by an FDA-approved test.^11,14^ In February 2021, tepotinib was approved in the United States, based on the results of a phase 2, single-arm, open-label trial (VISION; NCT02864992) that evaluated its safety, efficacy, and tolerability in patients, including those with locally advanced or metastatic stage IIIb/IV NSCLC harboring *MET*ex14 skipping.^9^ Other targeted therapies—such as crizotinib (administered off label in some contexts) and savolitinib (conditionally approved in China based on favorable phase 2 results)—have yet to be approved for this indication in the United States.^8,15-17^

The burden of mNSCLC on patients, caregivers, payers, and society and the recent emergence of targeted therapies that may prolong survival in the subset of patients whose tumors harbor *MET*ex14 skipping but may also result in additional treatment costs underscore the importance of developing evidence to assess both the health and cost outcomes of products within this class of treatments. This study aimed to support this endeavor by evaluating the cost-effectiveness of tepotinib from the US Medicare perspective, compared with capmatinib, for adults with mNSCLC harboring *MET*ex14 skipping.

## Methods

### Study Design and Scope

#### Overview of study design and scope

This cost-effectiveness analysis was based on the efficacy and safety results of the VISION study (cohort A; February 2021 data cutoff) for patients who were treatment naïve (1L) or previously treated (2L+), as per tepotinib’s FDA-approved indication.^9^ Capmatinib was included as the sole comparator to tepotinib, given that it is currently the only other targeted therapy approved for this indication in the United States. Although crizotinib may in certain contexts be administered for this indication, it was excluded from this analysis because it is not presently FDA approved, is not considered a preferred frontline or subsequent treatment option in current National Comprehensive Cancer Network Clinical Practice Guidelines,^18^ and, according to key opinion leaders consulted for this study, is unlikely to experience widespread utilization in the United States because of comparatively poor outcomes in clinical trials.^8,16^

The base-case analysis was performed from the US Medicare perspective and evaluated cost-effectiveness in 1L, 2L+, and line agnostic (1L and 2L+) settings. Deterministic sensitivity analysis (DSA) and probabilistic sensitivity analysis (PSA) and scenario analyses were performed to investigate the impact of parameter and structural uncertainty on model outcomes, including the effect of adopting commercial or Medicaid payer perspectives.

#### Model structure

**Figure 1.**
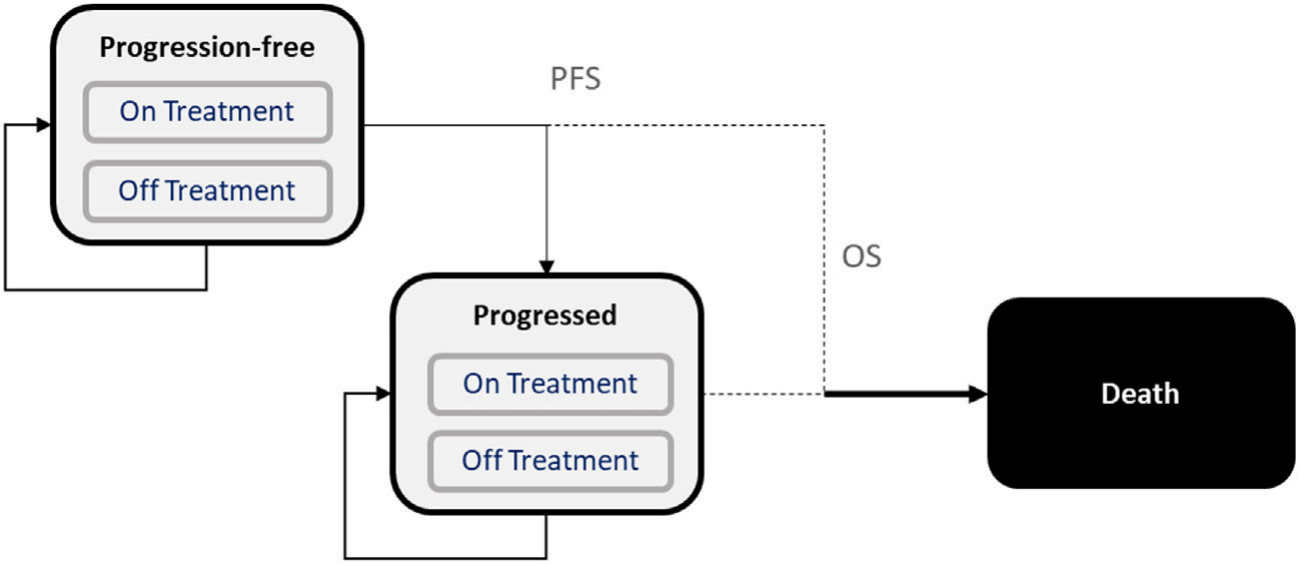
Model schematic. OS indicates overall survival; PFS, progression-free survival.

We developed a partitioned survival analysis model (PSM) in Microsoft Excel 2016 (Microsoft Corporation, Redmond, WA) that incorporated 3 health states, namely progression free, progressed, and deceased (Fig. 1). Overall survival (OS) curves governed mortality in the cohort, whereas progression-free survival (PFS) curves allocated those who remained alive during each cycle to progression-free and progressed disease states. Finally, time-to-discontinuation (TTD) curves dictated the proportion of patients undergoing treatment. A PSM rather than a Markov state transition model was developed because the PSM readily aligned with key secondary endpoints from VISION, simplified accounting for time dependency (achievable in state transition models through inclusion of tunnel states that contribute to structural complexity), and reduced reliance on unclassified endpoints to model state transitions.

Given that FDA approvals for tepotinib and capmatinib are not contingent upon treatment history, the cost-effectiveness model (CEM) was designed to capture costs and outcomes accrued by those who were 1L, 2L+, or either (ie, line agnostic). In the latter case, the CEM calculated the weighted average of outcomes for 1L and 2L+, with reference to the observed baseline distribution of patients in VISION (ie, 44.5% 1L, 55.5% 2L+),^9^ as opposed to explicitly modeling the patient journey across multiple lines of therapy. This approach provides greater flexibility in considering line-specific differences in treatment effects than building the model around the line agnostic intent-to-treat outcomes from the VISION study.

A 10-year time horizon was sufficient for nearly all patients to reach the death state. A monthly model cycle was selected, in alignment with dosing cycles, and a 3% annual discount rate was applied to health and cost outcomes.^19^

### Data Sources, Inputs, and Modeling

The CEM incorporated a wide range of clinical, utility, and cost/resource utilization inputs. A summary of key inputs used in the model and their respective sources is presented in Table 1.^9,10,20-35^

#### Clinical efficacy

Efficacy inputs for tepotinib were derived from the February 2021 data cutoff using the entire cohort A (n = 152) from VISION,^9^ consisting of patients who were 18 years of age or older with histologically or cytologically confirmed, locally advanced, or mNSCLC with *MET*ex14 skipping. Nearly all VISION participants (98%) had stage IV disease at study entry^9^; clinical experts advised that the remaining participants (ie, with stage IIIb disease who could not be treated with radiotherapy) were prognostically similar to patients with metastatic disease (of note, 99% of the GEOMETRY mono-1 efficacy population consisted of patients with stage IV disease).^36^ All patients had measurable disease, were able to perform most or all of the activities of daily living (ie, Eastern Cooperative Oncology Group score of 0 or 1), and had a negative test result for epidermal growth factor receptor mutations or anaplastic lymphoma kinase rearrangements.^9^

Standard parametric survival analysis techniques were applied to patient-level data from VISION to extrapolate PFS, OS, and TTD beyond the trial’s follow-up duration, in accordance with guidance issued by the Decision Support Unit for the National Institute for Health and Care Excellence.^37^ Briefly, this entailed identifying statistical distributions that exemplified goodness-of-fit (eg, evaluation of Akaike and Bayesian information criteria statistics and graphical assessment of fit versus observed data) and generated clinically plausible extrapolations (assessed via structured interviews with clinical experts). On this basis, exponential distributions were selected to model long-term OS and PFS for tepotinib, both for 1L and 2L+ patients. Details regarding the methodology and results of these statistical analyses are presented in the Supplemental Materials (Table 1 in Appendix 1 in Supplemental Materials found at https://doi.org/10.1016/j.jval.2022.11.018), whereas the impact of using alternative distributions was evaluated in scenario analyses.

Meanwhile, OS and PFS for capmatinib (Table 1 ^9,10,20-35^) were estimated using hazard ratios derived from an unanchored matching-adjusted indirect comparison (MAIC) study,^20^ using patient-level data from VISION and baseline variables and outcomes from GEOMETRY mono-1 (NCT02414139), a multi-cohort, open-label, phase 2 study designed to evaluate the safety and efficacy of capmatinib in patients with advanced NSCLC with *MET*ex14 skipping or *MET* amplification.^10^ To align with GEOMETRY mono-1 eligibility criteria and the efficacy population referenced in the current tepotinib US prescribing information, the MAIC focused on VISION cohort A participants identified by tissue biopsy (n = 88).

#### Treatment discontinuation

**Figure 2.**
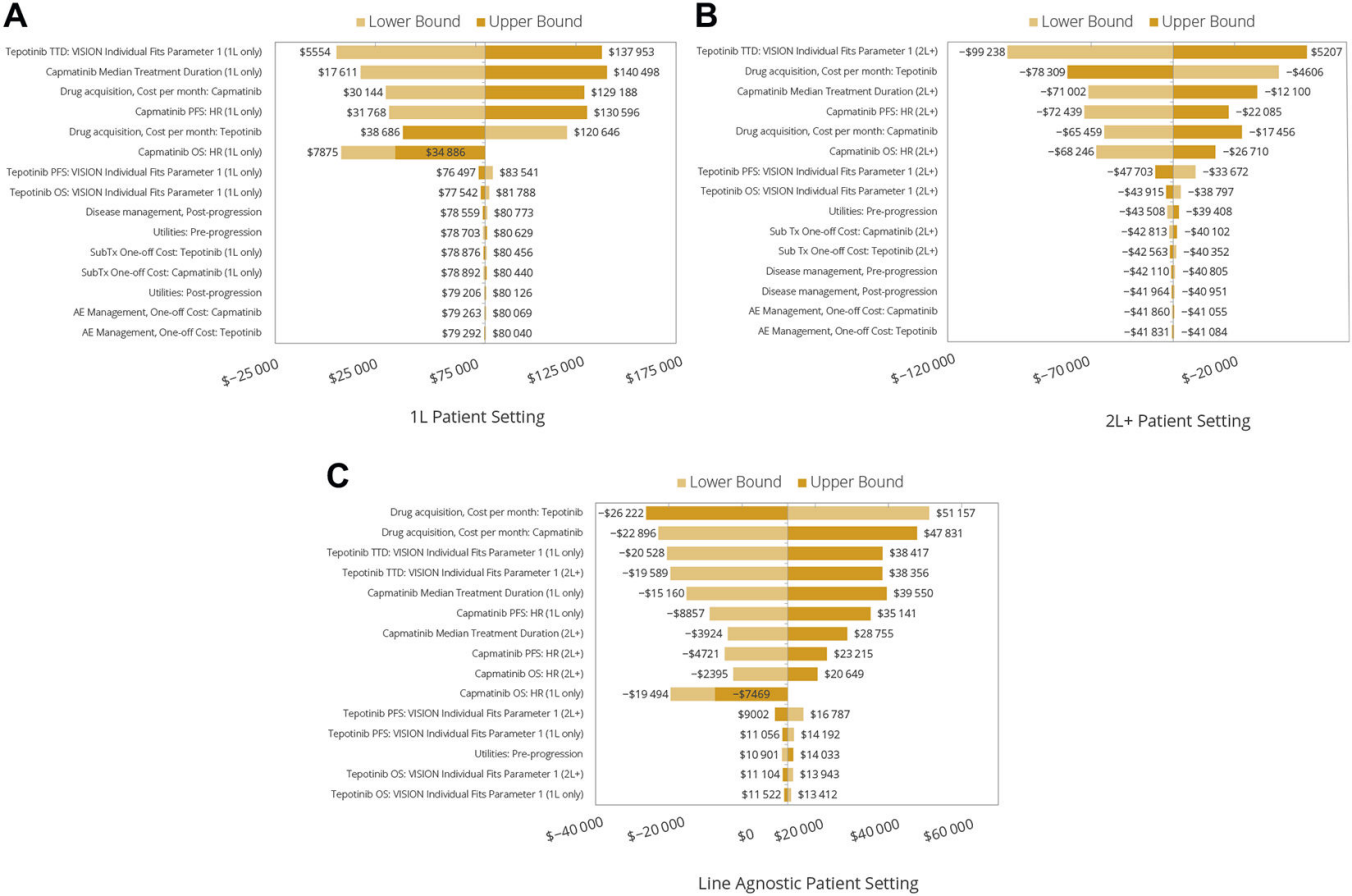
Deterministic sensitivity analysis (net monetary benefit) in each patient population: (A) 1L patient setting, (B) 2L+ patient setting, and (C) line agnostic patient setting. 1L indicates treatment naïve; 2L+, previously treated; AE, adverse event; HR, hazard ratio; OS, overall survival; PFS, progression-free survival; TTD, time to discontinuation; Tx, treatment. Note: incremental net monetary benefit is calculated as the value of incremental benefits associated with tepotinib (ie, incremental quality-adjusted life-years (QALYs), multiplied by the upper limit of the range of willingness-to-pay thresholds applied in this analysis [$150 000/QALY]), minus incremental costs.

**Figure 3.**
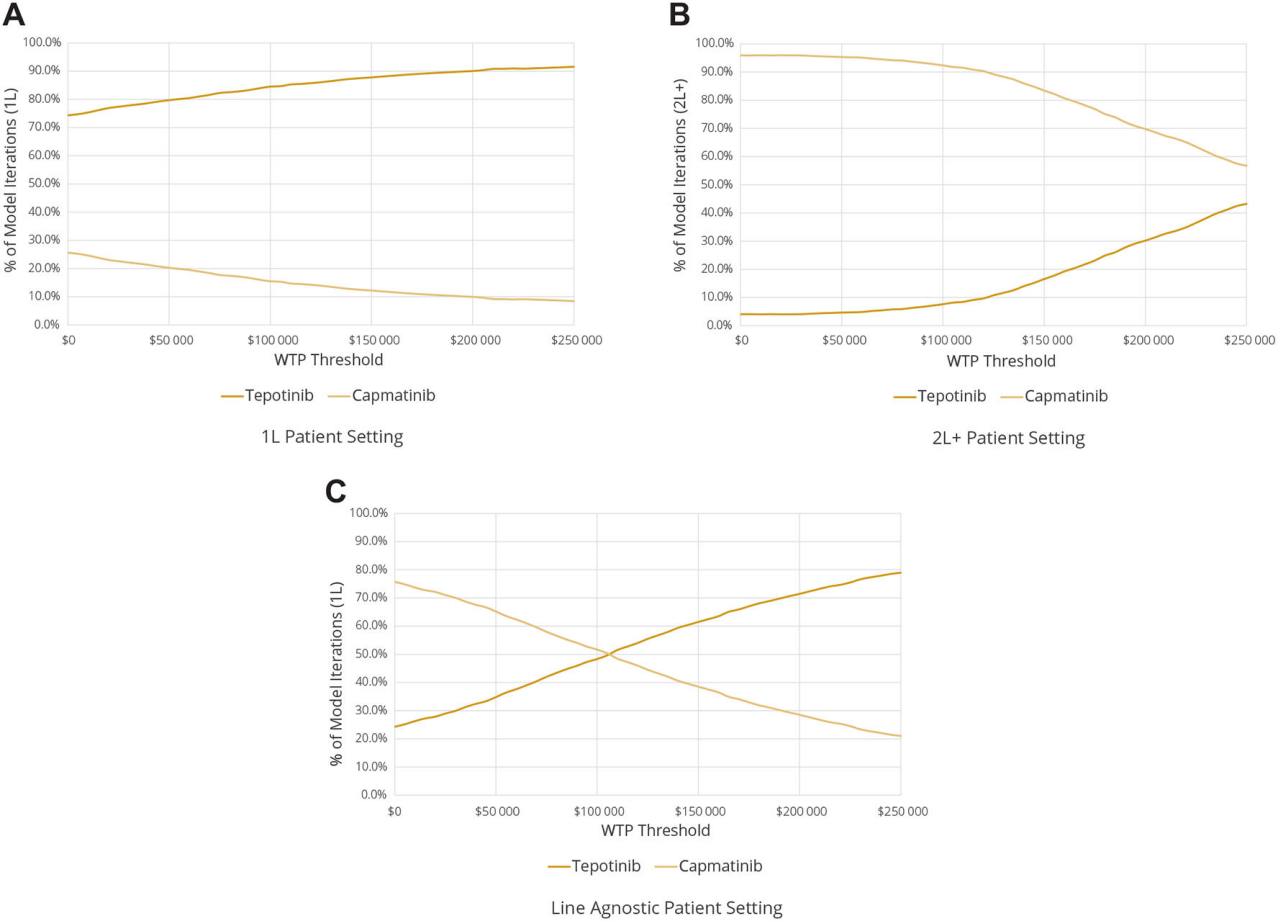
Cost-effectiveness acceptability curve for each patient population: (A) 1L patient setting, (B) 2L+ patient setting, and (C) line agnostic patient setting. 1L indicates treatment naïve; 2L+, previously treated; WTP, willingness to pay.

TTD in VISION was defined as months elapsed between the first and last doses of treatment and was extrapolated beyond the follow-up period by applying an exponential distribution (Figures 1-6 in Appendix 1 in the Supplemental Materials found at https://doi.org/10.1016/j.jval.2022.11.018). TTD for capmatinib was assumed to be the median duration of exposure reported in the GEOMETRY mono-1 study.^10^ This approach was applied to account for varying strategies around discontinuation, given that in clinical practice some patients discontinue treatment before progression, whereas others may continue until or past progression.

#### Costs and resource utilization

The CEM incorporated expenditures attributable to drug acquisition and administration, adverse event (AE) and disease management, treatment monitoring, and subsequent treatments (Appendices 2-5 in the Supplemental Materials found at https://doi.org/10.1016/j.jval.2022.11.018 for full sources and details). Biomarker testing costs were excluded from consideration in the base case, given that all patients entering the model were assumed to have confirmed *MET*ex14 skipping before treatment. All costs were inflated to 2021.^38^

Drug acquisition costs were based on dosing schedules in treatment labels and wholesale acquisition costs sourced from RED BOOK.^21^ Given that tepotinib and capmatinib are orally administered, recipients were assumed not to incur any drug administration costs. Subsequent treatment costs were applied to patients discontinuing either comparator and consisted of a one-off cost that accounted for post-tepotinib therapies administered in VISION, including TKIs (capmatinib or crizotinib if 2L+ with tepotinib, crizotinib if 2L+ with capmatinib), immuno-oncology monotherapy, immuno-oncology + chemotherapy ± antivascular endothelial growth factor, antivascular endothelial growth factor ± chemotherapy, and chemotherapy alone. Subsequent treatment costs were sourced like primary comparators and accrued for an interval estimated from mean PFS for subsequent therapy observed in VISION (3.0 months).^25^

For AE management costs, event incidence was drawn from product labels,^22,23^ whereas associated unit costs were procured from the Healthcare Cost and Utilization Project.^39^

Treatment monitoring was limited to laboratory costs, because it was assumed additional cost components were subsumed under disease management; based on input from clinical experts, monitoring was assumed similar for both treatments and consisted of monthly hematology and coagulation, liver function, electrolyte, and urine analysis testing, costs for which were sourced from the Centers for Medicare & Medicaid Services.^24^

Resources required for disease management were assumed to vary according to progression status. Estimates of medical resource utilization for NSCLC were based largely upon values obtained from Graham et al, 2016,^28^ and Dalal et al, 2018,^27^ whereas accompanying costs were sourced from the Centers for Medicare & Medicaid Services.^24,26^ Utilization of selected resources for patients with progressed disease (ie, specialist visits, computerized tomography scans, magnetic resonance imaging, ultrasounds, and X-rays) was derived from key opinion leader input. Unit costs and monthly frequencies were combined to obtain monthly disease management costs ($874 and $5462 per month for progression-free and progressed patients, respectively). Finally, one-off costs from published sources were accrued for disease progression ($1079) and terminal care ($4063).^29,30^

#### Health state utility values

Statistical analyses of EuroQol Five Dimension Five Level Scale data from VISION (cohort A) were undertaken to derive baseline, preprogression, and progressed utility values^31^ and were assumed equivalent for tepotinib and capmatinib. The negative effects of AEs on health-related quality of life were captured through utility decrements extracted from publicly available sources and estimated episode duration for each AE. Additional information regarding the health state utilities and utility decrements is supplied in Appendix 6 in the Supplemental Materials found at https://doi.org/10.1016/j.jval.2022.11.018.

### Model Validation and Analysis

A targeted literature review of economic models for mNSCLC was conducted at the model conceptualization phase to ensure the current CEM design would align with previous studies.^40-46^ The model validation process followed current guidelines from the ISPOR-Society of Medical Decision Making.^47^ The model structure, assumptions, and inputs were validated by clinical experts and experienced health economists. A technical validation of the model programming was performed by a modeler not previously involved in the study.

The base-case analysis assessed the cost-effectiveness of tepotinib versus capmatinib in terms of the incremental cost-effectiveness ratio (ICER), with health benefits quantified as quality-adjusted life-years (QALYs). Given that no explicit willingness-to-pay (WTP) threshold has been defined for the United States,^48^ results were interpreted with reference to the range of WTP thresholds recommended by the Institute for Clinical and Economic Review ($100 000-$150 000/QALY),^49^ as in other recently published health economic evaluations in NSCLC.^50^

DSA was conducted to probe uncertainty in model parameters by systematically adjusting the upper and lower bounds of individual parameters or groups thereof (using 95% confidence intervals, where available; otherwise, ±15% ranges around the point estimate) and examining the effect on model outcomes, presenting the results as a tornado diagram that ranks them in descending order according to impact. DSA results are expressed in terms of incremental net monetary benefit (INMB) and reflect the difference between incremental costs and the value of incremental QALYs (evaluated using a $150 000/QALY WTP threshold), with positive INMB estimates denoting cost-effectiveness of tepotinib relative to its comparator.

PSA was conducted to further examine the implications of parameter uncertainty by jointly and repeatedly sampling from suitable probability distributions defined for each parameter (summarized in Table 1 ^9,10,20-35^) and storing model outputs; 2500 iterations of the model were conducted in this fashion and convergence confirmed, and the results visualized through scatterplots as well as cost-effectiveness acceptability curves that illustrate the likelihood of achieving cost-effectiveness over a range of WTP thresholds.

Scenario analysis was conducted to quantify the cost-effectiveness of tepotinib versus capmatinib when key base-case assumptions were varied. A summary of each scenario and justification for its inclusion is presented in Table 6 in Appendix 7 in the Supplemental Materials found at https://doi.org/10.1016/j.jval.2022.11.018.

## Results

### Base-Case Analysis

In the 1L setting (Table 2), tepotinib and capmatinib generated 2.08 and 1.77 mean discounted life-years (LYs) (0.3076 incremental), respectively, and 1.44 and 1.22 mean discounted QALYs (0.2127 incremental). Patients treated with tepotinib or capmatinib accrued $347 719 and $395 475 in discounted lifetime costs, respectively, amounting to cost savings of $47 756; this was attributable largely to reduced drug acquisition costs ($56 947) and moderately offset by higher disease management costs ($9361), with other expenditures contributing only nominally to results. Thus, tepotinib was more effective and less costly than capmatinib in this setting and so considered to dominate its comparator.

In the 2L+ setting, tepotinib and capmatinib generated 2.08 and 1.61 mean discounted LYs (0.4666 incremental) and 1.41 and 1.08 mean discounted QALYs (0.3330 incremental), while accruing $338 520 and $247 119 in discounted lifetime costs, respectively ($91 401 incremental). Increased costs with tepotinib in this setting were attributable primarily to higher drug acquisition costs ($85 668) and to a lesser extent higher disease management costs ($7495); as in 1L, the contribution of other expenditure categories was negligible. These results culminated in an ICER of $274 514/QALY, which lies above the range of WTP thresholds endorsed by the Institute for Clinical and Economic Review ($100 000-$150 000/QALY).^49^

A line agnostic patient population (ie, a weighted average of 1L and 2L+) yielded 2.08 and 1.68 incremental Lys (0.3958 incremental) and 1.42 and 1.14 mean discounted QALYs (0.2794 incremental) for tepotinib and capmatinib, respectively, while accruing $342 615 and $313 168 in discounted lifetime costs ($29 447 incremental). This culminated in an ICER of $105, 383/QALY, which lies well within the range of WTP thresholds endorsed by the Institute for Clinical and Economic Review.

### Sensitivity Analysis

#### Deterministic sensitivity analysis

Results of the DSA (Fig. 2) show that, in all patient populations, estimated INMB is most sensitive to monthly drug acquisition costs for both tepotinib and capmatinib, the parameters of the exponential distribution used to extrapolate TTD with tepotinib beyond trial follow-up, capmatinib median treatment duration, and hazard ratios for PFS and OS with capmatinib (detailed tabular results are presented in Tables 8, 9 and 10 in Appendix 8 in the Supplemental Materials found at https://doi.org/10.1016/j.jval.2022.11.018). Considering the range of WTP thresholds applied in this study, tepotinib was consistently cost-effective versus capmatinib in the 1L setting, but not in the 2L+ setting, whereas in a line agnostic context, results exhibited strong sensitivity to the cost of treatment with, and comparative efficacy of, tepotinib and capmatinib.

#### Probabilistic sensitivity analysis

In the 1L setting, tepotinib dominated capmatinib when mean incremental costs (−$47 367) and QALYs (0.2250) were considered. The probability of obtaining an ICER below $150 000/QALY was approximately 87.8% (Fig. 3). Because tepotinib typically yielded positive incremental QALYs and cost savings, it was more effective than capmatinib in 90.6% of iterations, less costly in 74.4% of iterations, and dominant 66.5% of the time. In the 2L+ setting, by contrast, the PSA produced a mean ICER of $282 450/QALY (incremental mean costs: $94 455; incremental mean QALYs: 0.3344), and the likelihood of cost-effectiveness at a WTP threshold of $150 000 was estimated at 16.6%. Tepotinib accrued both higher costs and QALYs in 86.3% of model iterations. Finally, in the line agnostic setting, the mean ICER was $108 902/QALY (incremental mean costs: $31 236; incremental mean QALYs: 0.2868), whereas the probability of cost-effectiveness at a WTP threshold of $150 000/QALY was 61.5%. Tepotinib was usually more costly and more effective than capmatinib in this setting (72.9% of iterations) and was dominant 21.4% of the time.

#### Scenario analysis

Scenario analyses are presented in Table 3 (detailed tabular results are presented in Table 7 in Appendix 7 in the Supplemental Materials found at https://doi.org/10.1016/j.jval.2022.11.018). Assuming time on treatment was equal to PFS had the greatest impact on model outcomes in 1L, 2L+, and line agnostic patient populations, given that PFS translated into significantly longer duration of treatment with tepotinib (average of 17.1 vs 13.2 months in 1L; 13.4 vs 11.8 in 2L+) and thus higher lifetime costs, whereas the opposite was true for 1L capmatinib (average of 14.6 vs 16.5 months in 1L; 8.3 vs 7.9 in 2L+). In the 1L setting, tepotinib’s dominance over capmatinib was sustained in all remaining scenarios, whereas in 2L+, ICERs consistently exceeded the $100 000 to $150 000/QALY range of WTP thresholds. ICERs for alternative scenarios in the line agnostic context tended to cluster around the base-case value, although adopting a commercial perspective produced a marked increase in the ICER, whereas assuming a larger proportion of frontline patients or applying alternative distributions for the tepotinib PFS curves typically had the opposite effect.

## Discussion

This study evaluates the cost-effectiveness of tepotinib versus capmatinib in adult patients with mNSCLC harboring *MET*ex14 skipping from the perspective of US Medicare payers over a lifetime horizon. To support the analysis, we constructed a de novo 3-state PSM, which we then populated with inputs derived from the February 2021 data cutoff of the phase 2 VISION trial (cohort A), an unanchored MAIC, US costing databases, published literature, and input from clinical experts.

In the reference case, tepotinib in the 1L setting was associated with 0.2127 incremental discounted QALYs and a discounted cost savings of $47 756 over the model horizon, thereby dominating capmatinib. In 2L+, the simulated patient cohort treated with tepotinib accrued 0.3330 more QALYs and $91 401 in incremental costs, culminating in an ICER of $274 514/QALY, which lies above the range of values endorsed by the Institute for Clinical and Economic Review ($100 000-$150 000/QALY).^49^ In a line agnostic patient population, tepotinib yielded 0.2794 additional QALYs and $29 447 in incremental costs versus capmatinib, resulting in an ICER of $105 383/QALY that lies well within the range of WTP thresholds used in this analysis.

Differences in outcomes across treatment lines are largely attributable to estimates of treatment duration. In 1L, mean treatment durations for tepotinib and capmatinib were 13.2 months and 16.5 months, respectively, whereas in 2L+, the average treatment durations were 11.8 months and 7.9 months, respectively. With capmatinib having a longer time on treatment in 1L, additional monthly drug acquisition costs are accrued, and these account for a significant portion (approximately 70%) of the total costs associated with each treatment.

The DSA, PSA, and scenario analyses for the 1L and 2L+ settings tended to align with their respective base-case results. By contrast, the cost-effectiveness of tepotinib in the line agnostic context was highly sensitive to uncertainty in drug acquisition costs and efficacy inputs in the DSA and to analytical perspective (eg, higher ICERs with a commercial perspective due to increased disease management unit costs, coupled with increased OS with tepotinib). It was also sensitive to the composition of the patient population (which produced favorable ICERs when applying population weighting from Flatiron,^51^ in which a larger proportion of patients were in 1L [1L, 56.4%; 2L+, 44.4%] than VISION [1L, 44.5%; 2L+, 55.5%]), and the choice of distributions used to extrapolate PFS for tepotinib beyond the trial horizon. Assuming time on treatment equal to PFS yielded higher ICERs in all patient populations because time to progression was markedly higher than TTD for tepotinib, whereas the opposite was true for capmatinib in the 1L setting (PFS exceeded TTD for both comparators in 2L+). PSA results in the line agnostic patient population imply that tepotinib is likely to exhibit cost-effectiveness over capmatinib for most plausible combinations of input values, but also suggest results should be interpreted with caution because of uncertainty across a range of key model parameters.

At the time of writing, this is the first published health economic evaluation of TKIs in adults with mNSCLC harboring *MET*ex14 skipping and therefore offers a valuable contribution to the emerging understanding of health and cost outcomes associated with tepotinib and capmatinib in this patient population. We used a modeling methodology that is common in oncology and consistent with best practice as laid out by ISPOR-Society of Medical Decision Making,^47^ and the model was thoroughly reviewed and validated by clinical experts.

It is essential to recognize this study’s limitations and short-comings in interpreting its results. First, no head-to-head studies of tepotinib versus capmatinib have been undertaken to date; accordingly, an unanchored MAIC was conducted to account for differences in trial populations, focusing on key prognostic differences to preserve sample size.^52^ This approach is preferable to naïve side-by-side comparisons of outcomes between clinical trials because it reduces bias that may occur because of differences in baseline demographic and clinical characteristics between the trial populations.^52^ Nevertheless, the absence of a common comparator in the VISION and GEOMETRY mono-1 trials necessitates reliance on assumptions that may not hold in practice; for instance, this approach assumes the analysis has captured all relevant effect modifiers and prognostic factors, which is generally unlikely to be achieved, and may introduce bias into the results.^53^ In addition, small sample sizes contributed to wide confidence intervals around key efficacy inputs and, by extension, to the uncertainty observed in the sensitivity and scenario analysis results.

Second, the analysis did not include crizotinib, another targeted therapy used in some instances to treat this indication. Although this choice was seen as appropriate given the scope of the analysis (ie, targeted therapies FDA approved for this indication in the United States) and supported by current National Comprehensive Cancer Network guidelines and input from clinical experts, the authors recognize this as a limitation of the study to the extent it implies excluding an intervention that may represent a relevant therapeutic alternative in certain contexts.

Third, as is common in health economic evaluations in oncology, it was necessary to extrapolate beyond the comparatively brief trial follow-up duration, introducing further uncertainty into the results. To mitigate this uncertainty, we applied standard parametric survival analysis techniques to patient-level data from VISION in accordance with current best practice,^37^ ultimately concluding that exponential distributions were appropriate for long-term extrapolation of OS, PFS, and TTD to the extent they exemplified both statistical goodness-of-fit and clinical plausibility. This was further investigated in scenario analyses, which determined that adopting alternative distributions for OS and PFS does not materially alter the central findings of this study.

Fourth, plausible assumptions and expert opinion were applied to address outstanding data gaps. For example, there is an absence of evidence specific to mNSCLC harboring *MET*ex14 skipping, and therefore, wild-type mNSCLC published studies were used to inform several model inputs, although this is not expected to bias the results of the study in favor of or against, tepotinib. Additionally, although VISION and GEOMETRY mono-1 recruited patients with both advanced (stage IIIb) and metastatic (stage IV) tumors, patients with stage IIIb disease comprised a very small proportion of participants in both trials^9,36^ and in VISION, because they could not be treated with radiotherapy, were considered prognostically similar to patients with stage IV disease. Accordingly, clinical inputs derived from both trials were assumed representative of patients with mNSCLC.

## Conclusions

Tepotinib may be cost-effective compared with capmatinib in treating patients with mNSCLC harboring *MET*ex14 skipping in frontline and line agnostic settings from the US Medicare perspective; in subsequent lines, cost-effectiveness depends upon the value payers attach to additional QALYs. These results are subject to uncertainty and should be revisited as the evidence base for this class of therapies matures.

## Supplementary Material

1

## Figures and Tables

**Table 1.** Inputs for the reference case.

Input		Deterministic value		Probability distributions for PSA		Reference
		1L	2L+	1L	2L+	
Clinical efficacy*						
OS in months, tepotinib (mean)		26.9	27.0	Correlated draws from multivariate normal distributions from Cholesky decomposition of covariance matrices		VISION analysis^9^; MAIC with prognostic variables adjusted^20^
OS, capmatinib HR vs tepotinib		1.19	1.32	Lognormal^†^; μ = −0.20; σ = 0.15	Lognormal^†^; μ = −0.43; σ = 0.15	
OS in months, capmatinib (mean)		22.7	20.6	N/A (estimated by applying HR to exponential distribution to VISION survival data)		
PFS in months, tepotinib (mean)		17.1	13.4	Correlated draws from multivariate normal distributions from Cholesky decomposition of covariance matrices		
PFS, capmatinib HR vs tepotinib		1.18	1.67	Lognormal^†^; μ = −0.36; σ = 0.41	Lognormal^†^; μ = −0.69; σ = 0.38	
PFS in months, capmatinib (mean)		14.6	8.3	N/A (estimated by applying HR to exponential distribution to VISION survival data)		
TTD in months, tepotinib (mean)		13.2	11.8	Correlated draws from multivariate normal distributions from Cholesky decomposition of covariance matrices		VISION analysis^9^
TTD in months, capmatinib	Mean	16.5	7.9	N/A*		Wolf (duration of exposure as proxy)^10^
	Median	11.1	5.1	Gamma; α = 96.04; β = 0.12	Gamma; α = 96.04; β = 0.05	
Drug acquisition						
Drug acquisition cost (WAC), tepotinib		$20 899		N/A		IBM^21^
Drug acquisition cost (WAC), capmatinib		$9469		N/A		
Cost per month, tepotinib		$21 131		Gamma; α = 170.73; β = 123.77		Calculation
Cost per month, capmatinib		$20 515		Gamma; α = 170.73; β = 120.16		
Unit size, tepotinib		225 mg		N/A		EMD Serono; FDA labels^22,23^
Unit size, capmatinib		200 mg		N/A		
Unit per box, tepotinib		60 tablets		N/A		
Unit per bottle, capmatinib		56 tablets		N/A		
Drug dosing details, tepotinib		450 mg		N/A		
Drug dosing details, capmatinib		400 mg		N/A		
Frequency, tepotinib		Once daily		N/A		
Frequency, capmatinib		Twice daily		N/A		
Subsequent treatment costs						
One-off cost, tepotinib		$14 428		Gamma; α = 170.73; β = 84.51	Gamma; α = 170.73; β = 84.51	CMS.gov^24^; IBM^21^; KOL feedback; VISION CSR^25^
One-off cost, capmatinib		$14 335		Gamma; α = 170.73; β = 83.96	Gamma; α = 170.73; β = 83.96	
DM and treatment monitoring costs						
DM: preprogression (per cycle)		$874		Gamma; α = 170.73; β = 5.12		CMS.gov^26^; Dalal et al^27^; Graham et al^28^; KOL feedback
DM: postprogression (per cycle)		$5462		Gamma; α = 170.73; β = 31.99		
Disease progression (one off)		$1079		Gamma; α = 170.73; β = 6.32		Georgieva et al^29^
Terminal care (one off)		$4063		Gamma; α = 170.73; β = 23.80		Chastek et al^30^
Treatment monitoring, tepotinib (per cycle)		$25		Gamma; α = 170.73; β = 0.15		CMS.gov^26^; KOL feedback
Treatment monitoring, capmatinib (per cycle)		$25		Gamma; α = 170.73; β = 0.15		
Utility weights						
Progression free		0.72		Beta; α = 512.58; β = 197.07		VISION trial^31^
Progressed disease		0.63		Beta; α = 760.83; β = 438.84		
AE management						
Total disutility due to grade 3-4		−0.0010		Beta; α = 170.56; β = 172 406.20		ICER 2016^32^; NICE
AEs (one-off decrement), tepotinib						TA578^33^
Total disutility due to grade 3-4		−0.0015		Beta; α = 170.48; β = 114 655.50		
AEs (one-off decrement), capmatinib						
Total grade 3-4 AE costs (one off), tepotinib		$2492		Gamma; α = 170.73; β = 14.60		CMS.gov^26^; VISION CSR^25^; FDA labels^22,23^;
Total grade 3-4 AE costs (one off), capmatinib		$2685		Gamma; α = 170.73; β = 15.73		Shimizu et al^34^; Patel et al^35^

1L indicates treatment naïve; 2L+, previously treated; AE, adverse event; CSR, Clinical Study Report; DM, disease management; FDA, Food Drug Administration; HR, hazard ratio; ICER, Institute for Clinical and Economic Review; KOL, key opinion leader; MAIC, matching-adjusted indirect comparison; N/A, not available; NICE, The National Institute for Health and Care Excellence; OS, overall survival; PFS, progression-free survival; PSA, probabilistic sensitivity analysis; TTD, time to discontinuation; WAC, wholesale acquisition cost.

* Efficacy inputs for tepotinib (OS, PFS, and TTD) were derived from parametric survival analyses of VISION data whereas HRs for capmatinib OS and PFS were derived from an MAIC undertaken using published data from GEOMETRY mono-1 and the tissue biopsy only subgroup of VISION cohort A; published estimates of median duration of exposure proxied for capmatinib TTD. Mean values are calculated and presented in the table to facilitate comparability.

† These represent log-scale estimates of the mean and standard error of the original HR, which was expressed in terms of tepotinib versus capmatinib. In the PSA, these inputs were derived by exponentiating the random draw obtained from the normal distribution using the log-scale parameters and then calculating the inverse.

**Table 2.** Base-case analysis deterministic results.

	1L		2L+		Overall (line agnostic)	
	Tepotinib	Capmatinib	Tepotinib	Capmatinib	Tepotinib	Capmatinib
Health outcomes						
*Total QALYs*	1.4363	1.2236	1.4121	1.0791	1.4229	1.1435
Progression-free LYs	1.3465	1.1515	1.0567	0.6416	1.1857	0.8686
Postprogression LYs	0.7328	0.6202	1.0247	0.9731	0.8947	0.8160
On-treatment LYs	1.0365	1.3004	0.9278	0.6086	0.9762	0.9166
Off-treatment LYs	1.0428	0.4713	1.1535	1.0062	1.1042	0.7681
*Total LYs*	2.0793	1.7717	2.0814	1.6148	2.0804	1.6847
Cost outcomes						
Drug acquisition, $	273 198	330 145	245 679	160 012	257 931	235 756
Administration, $	0	0	0	0	0	0
Treatment monitoring, $	312	392	280	183	294	276
Adverse event management, $	2492	2685	2492	2685	2492	2685
Disease management, $	66 451	57 090	82 697	75 202	75 464	67 139
Subsequent treatment, $	5265	5163	7372	9037	6434	7312
*Total costs*, $	347 719	395 475	338 520	247 119	342 615	313 168
Incremental results						
Incremental costs, $	–	−47 756	–	91 401	–	29 447
Incremental LYs	–	0.3076	–	0.4666	–	0.3958
Incremental QALYs	–	0.2127	–	0.3330	–	0.2794
ICER ($/LY)	–	Dominant	–	195 904	–	74 404
ICER ($/QALY)	–	Dominant	–	274 514	–	105 383

1L indicates treatment naïve; 2L+, previously treated; ICER, incremental cost-effectiveness ratio; LY, life-year; QALY, quality-adjusted life-year.

**Table 3.** List of scenarios and results.

Scenario description	ICER/QALY		
	1L, $	2L+, $	Line agnostic, $
Base-case analysis	Dominant	274 514	105 383
Assume treat until progression	317 373	348 208	337 757
Include biomarker testing costs	Dominant	274 514	105 383
Use alternative DM resource utilization	Dominant	267 071	97 208
Exclude subsequent treatment expenditures	Dominant	279 514	108 525
Double subsequent treatment frequencies	Dominant	269 514	102 241
Literature-based PF and PD utility values	Dominant	310 371	120 893
Exclude AE disutilities	Dominant	274 924	105 571
5-year time horizon	Dominant	314 710	114 881
20-year time horizon	Dominant	270 284	105 173
5% cost and health outcomes discount rates	Dominant	276 329	107 202
0% cost and health outcomes discount rates	Dominant	260 786	102 157
Commercial perspective	Dominant	318 644	156 864
Medicaid perspective	Dominant	273 238	108 740
Apply population weighting from Flatiron	N/A	N/A	52 221
Apply $35 copayment	Dominant	275 108	106 989
Apply 10% coinsurance	Dominant	249 276	97 756
Weibull distribution for tepotinib OS curve	Dominant	321 202	103 734
Lognormal distribution for tepotinib OS curve	Dominant	255 890	104 627
Log-logistic distribution for tepotinib OS curve	Dominant	256 241	104 450
Gamma distribution for tepotinib OS curve	Dominant	272 522	104 449
Gompertz distribution for tepotinib OS curve	Dominant	325 501	104 462
Weibull distribution for tepotinib PFS curve	Dominant	285 014	110 921
Lognormal distribution for tepotinib PFS curve	Dominant	254 715	86 027
Log-logistic distribution for tepotinib PFS curve	Dominant	244 832	81 320
Gamma distribution for tepotinib PFS curve	Dominant	242 164	78 973
Gompertz distribution for tepotinib PFS curve	Dominant	260 027	87 182

1L indicates treatment naïve; 2L+, previously treated; AE, adverse event; DM, disease management; ICER, incremental cost-effectiveness ratio; N/A, not available; OS, overall survival; PD, progressed disease; PF, progression free; PFS, progression-free survival; QALY, quality-adjusted life-year.
