# Supplementary material for: Cost-Effectiveness of Tepotinib Versus Capmatinib for the Treatment of Adult Patients With Metastatic Non–Small Cell Lung Cancer Harboring Mesenchymal–Epithelial Transition Exon 14 Skipping
Source: Value Health. Author manuscript; Available in PMC 2023 Aug 14. (PMC10424058; doi:10.1016/j.jval.2022.11.018)
Supplement: 1 [file NIHMS1920822-supplement-1.docx]

Cost-Effectiveness of Tepotinib versus Capmatinib for the Treatment of Adult Patients with Metastatic Non-Small Cell Lung Cancer Harboring Mesenchymal–epithelial Transition Exon 14 (*MET*ex14) Skipping

# Supplemental Material

## Appendix 1. Parametric Fits for Tepotinib

The process of selecting a best fitting distribution involved statistical goodness of fit and clinical considerations (plausibility of results). Statistical goodness of fit was assessed using Akaike Information Criterion (AIC) and Bayesian Information Criterion (BIC) statistics and graphical assessment of fit versus observed data^1^ (Table 1).

Assessment also relied on clinical judgment of plausibility of extrapolations, which was evaluated by examining the shape of the long-term projection and predicted median, mean, and long-term survival (the 99% quantile of the fitted distribution, i.e., time after which less than 1% of the patients is event free). The above-mentioned statistics^1^ were presented to key opinion leader (KOL) clinicians from the US and Canada, who were asked to validate the choice of distribution from the list of fitted parametric distributions to be used in the base case and scenario analyses. The exponential distribution was selected as the preferred choice for overall survival (OS), progression-free survival (PFS), and time-to-discontinuation (TTD) for tepotinib in the base-case analysis. Alternative distributions were considered in scenario analyses.

**Table 1.** Statistical Goodness of Fit (AIC/BIC) for Selected Parametric Distributions.

|  | OS for 1L Tepotinib | | OS for 2L+ Tepotinib | | PFS for 1L Tepotinib | | PFS for 2L+ Tepotinib | | TTD for 1L Tepotinib | | TTD for 2L+ Tepotinib | | |
| --- | --- | --- | --- | --- | --- | --- | --- | --- | --- | --- | --- | --- | --- |
| **Distribution** | **AIC** | **BIC** | **AIC** | **BIC** | **AIC** | **BIC** | **AIC** | **BIC** | **AIC** | **BIC** | **AIC** | **BIC** |  |
| Weibull | 337.1 | 341.6 | 410.9 | 415.7 | 339.3 | 343.7 | 451.5 | 456.3 | 414.1 | 418.5 | 516.5 | 521.3 |  |
| Lognormal | 337.4 | 341.8 | 409.7 | 414.5 | 334.3 | 338.8 | 444.0 | 448.8 | 417.8 | 422.3 | 513.2 | 518.0 |  |
| Log-logistic | 336.2 | 340.7 | 410.3 | 415.1 | 334.1 | 338.6 | 445.9 | 450.7 | 411.8 | 416.2 | 514.9 | 519.7 |  |
| Exponential | 335.4 | 337.6 | 412.4 | 414.8 | 337.3 | 339.5 | 450.4 | 452.8 | 412.5 | 414.7 | 515.7 | 518.1 |  |
| Gamma | 338.3 | 345.1 | 411.4 | 418.6 | 336.3 | 343.0 | 445.8 | 453.0 | 414.9 | 421.6 | 514.6 | 521.9 |  |
| Gompertz | 337.4 | 341.9 | 413.2 | 418.1 | 337.3 | 341.8 | 452.0 | 456.8 | 412.1 | 416.6 | 517.6 | 522.5 |  |

AIC, Akaike Information Criterion; BIC, Bayesian Information Criterion; OS, overall survival; PFS, progression-free survival; TTD, time to discontinuation.

**Figure 1.** Comparison of Statistical Fits vs. Observed Data – Tepotinib 1L OS.


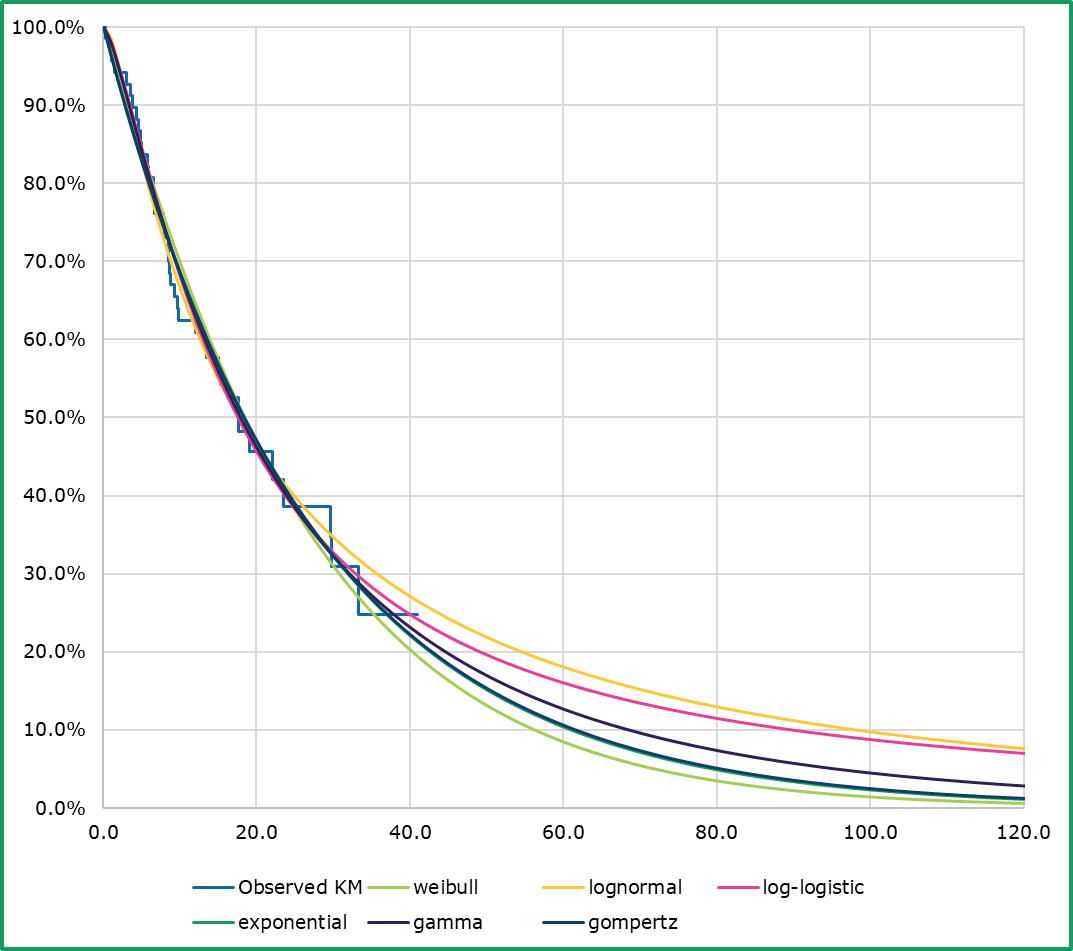


1L, first line; KM, Kaplan-Meier; OS, overall survival.

**Figure 2.** Comparison of Statistical Fits vs. Observed Data – Tepotinib 2L+ OS.


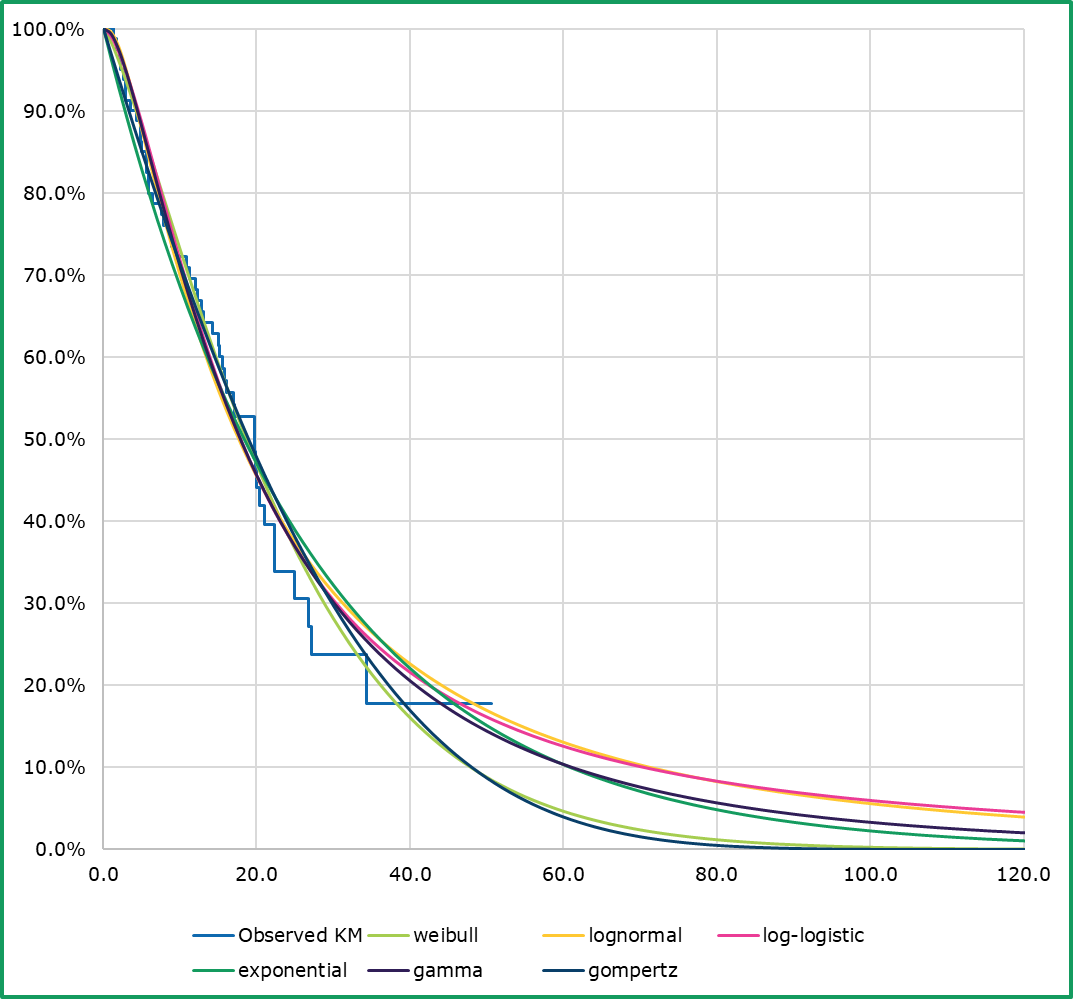


2L, second line; KM, Kaplan-Meier; OS, overall survival.

**Figure 3.** Comparison of Statistical Fits vs. Observed Data – Tepotinib 1L PFS.


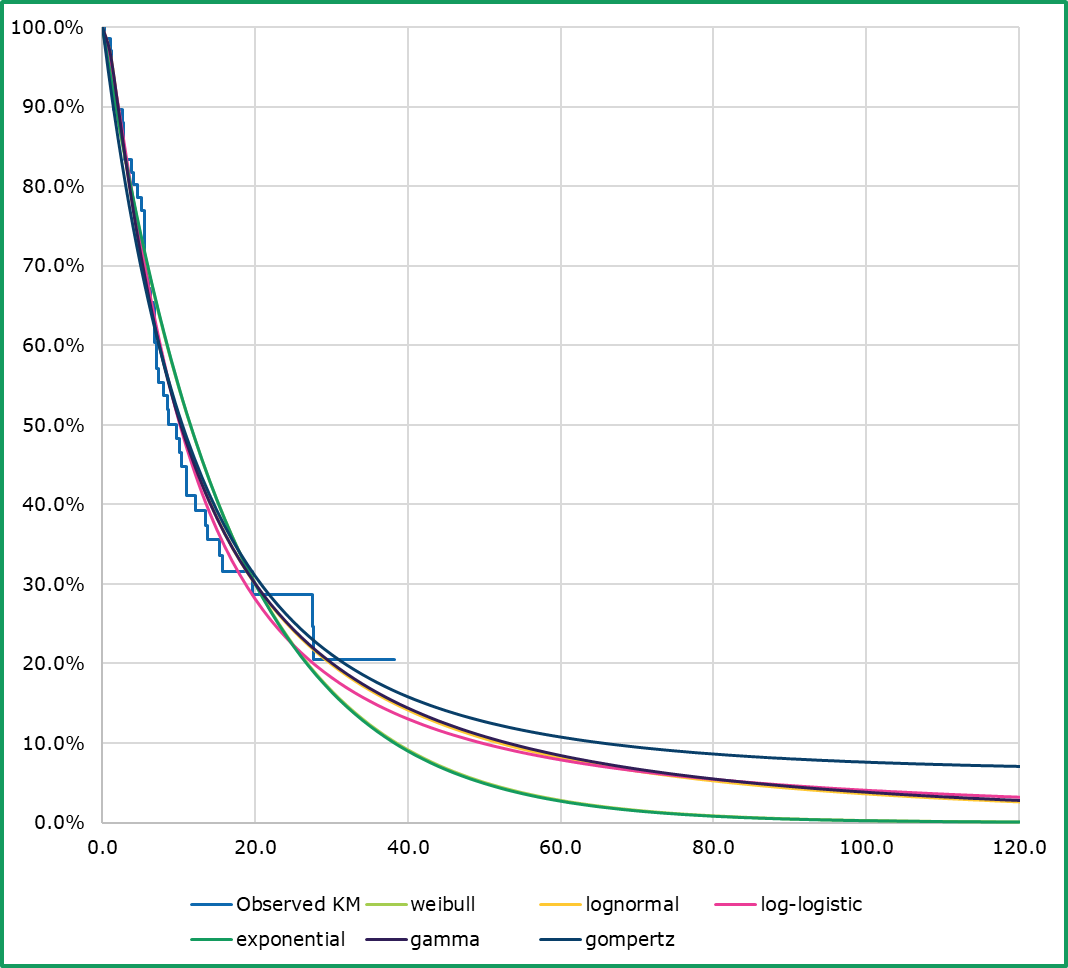


1L, first line; KM, Kaplan-Meier; PFS, progression-free survival.

**Figure 4.** Comparison of Statistical Fits vs. Observed Data – Tepotinib 2L+ PFS.


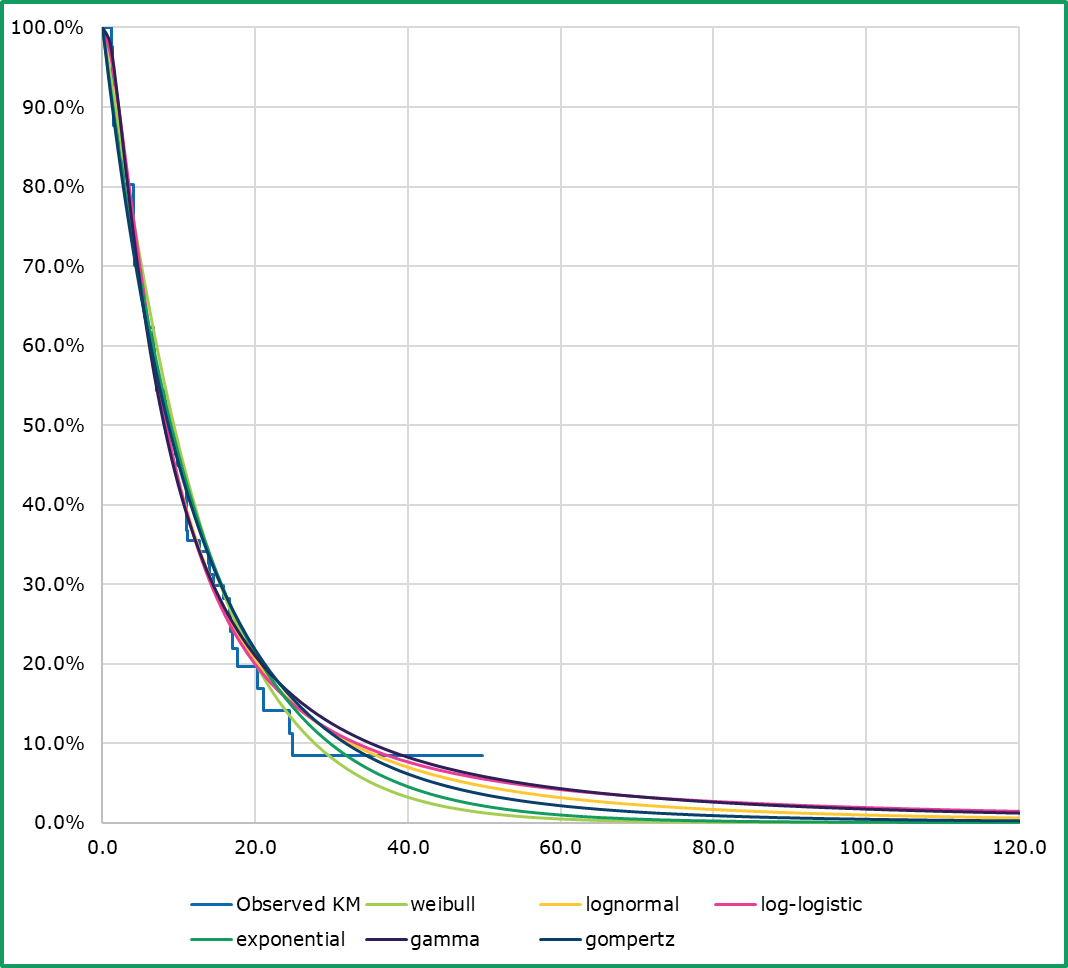


2L, second line; KM, Kaplan-Meier; PFS, progression-free survival.

**Figure 5.** Comparison of Statistical Fits vs. Observed Data – Tepotinib 1L TTD.


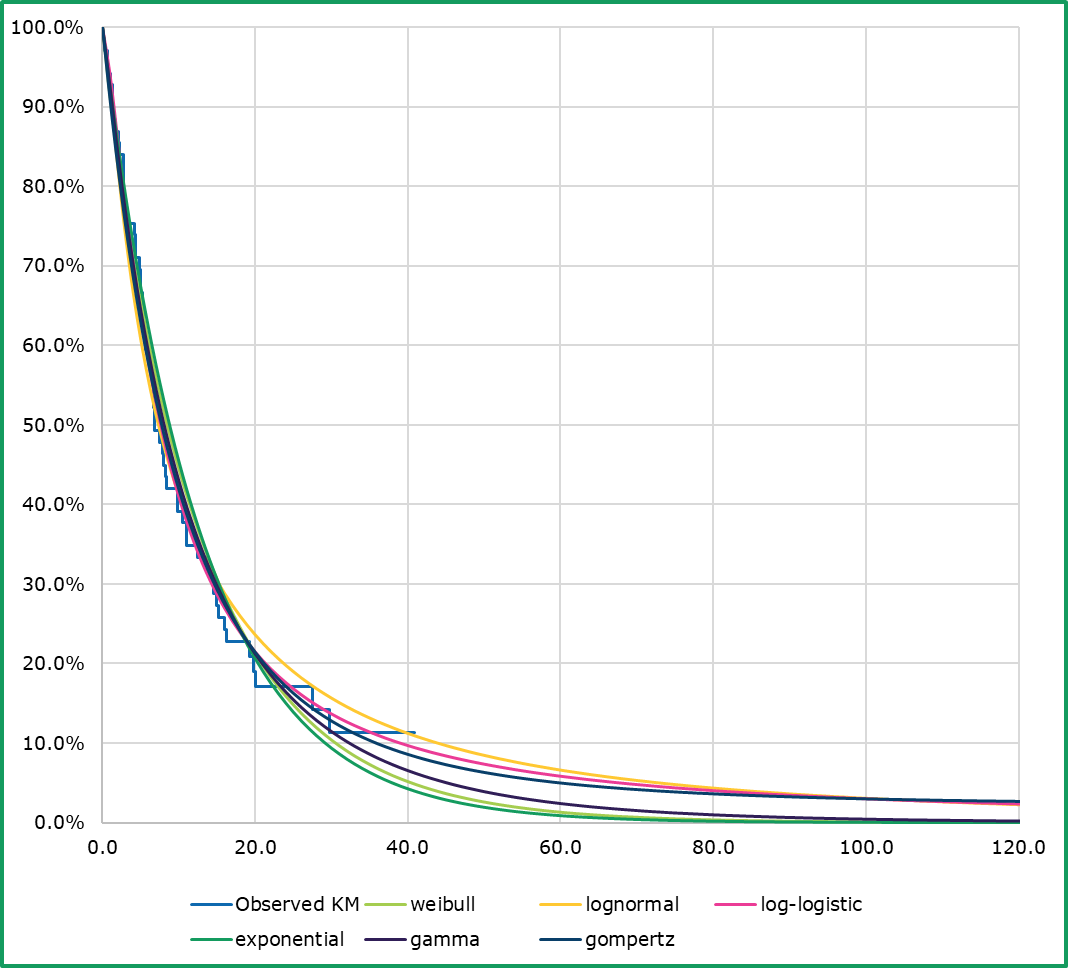


1L, first line; KM, Kaplan-Meier; TTD, time to discontinuation.

**Figure 6**. Comparison of Statistical Fits vs. Observed Data – Tepotinib 2L+ TTD.


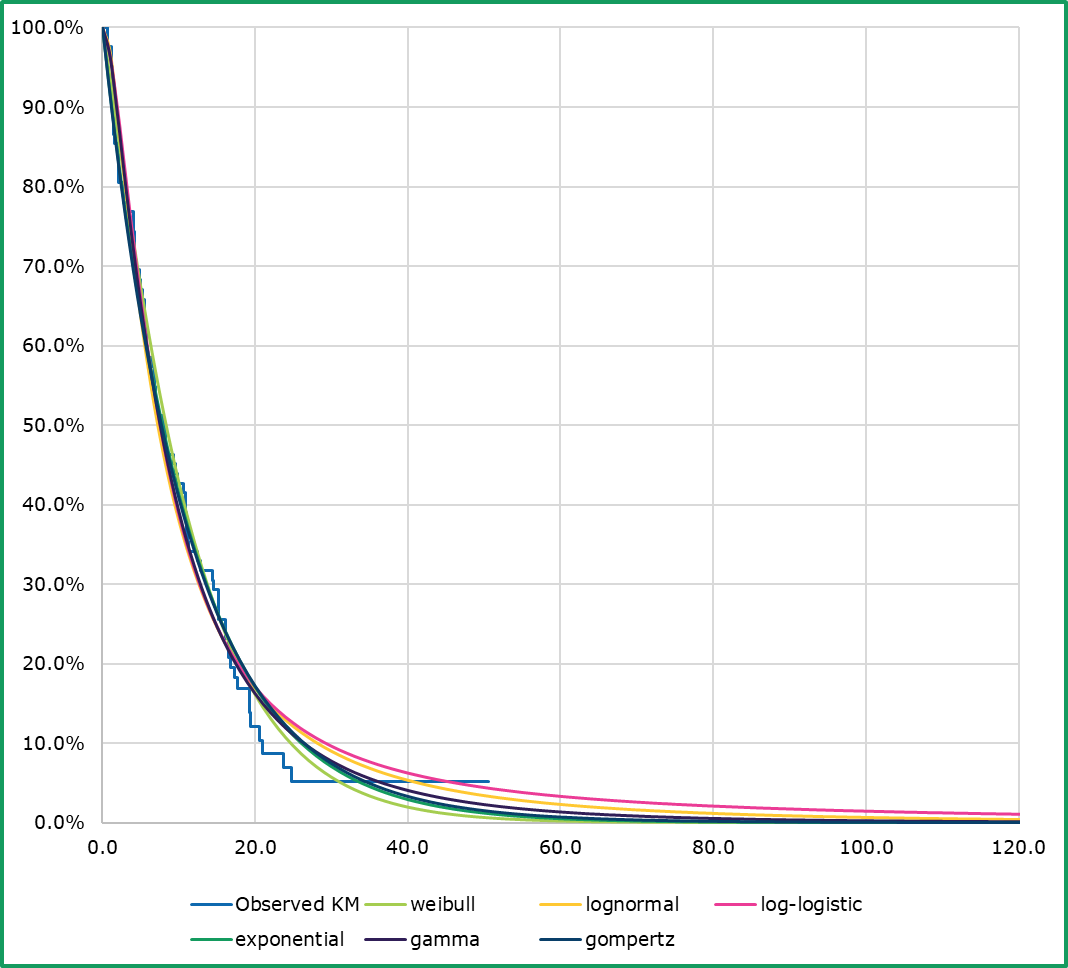


2L, second line; KM, Kaplan-Meier; TTD, time to discontinuation.

## Appendix 2. Tabular Summary of Disease Management Cost Calculations

**Table 2.** Disease Management Cost Calculations

|  | **Frequency per year** | | **Unit Cost: Medicare** | | | | **Unit Cost: Commercial** | | | | | | | **Unit Cost: Medicaid** | | | | | | |
| --- | --- | --- | --- | --- | --- | --- | --- | --- | --- | --- | --- | --- | --- | --- | --- | --- | --- | --- | --- | --- |
|  | **Pre-progression** | **Post-progression** | **Cost per intervention** | **Monthly cost** | | | | **Cost per intervention** | | | **Monthly cost** | | | | **Cost per intervention** | | | **Monthly cost** | | |
|  |  |  |  | **Pre-progression** | **Post-progression** | | |  |  |  | **Pre-progression** | **Post-progression** | | |  |  |  | **Pre-progression** | | **Post-progression** |
| GP visit | 0.047 | 3.050 | $56.88 | $2.68 | | $173.48 | | $103.00 | | $4.86 | | | $314.15 | | $52.76 | | $2.49 | | $160.92 | |
| Specialist visit | 0.500 | 1.000 | $131.20 | $65.60 | | $131.20 | | $215.00 | | $107.50 | | | $215.00 | | $120.20 | | $60.10 | | $120.20 | |
| Nurse visit | 0.129 | 0.117 | $23.03 | $2.98 | | $2.69 | | $61.00 | | $7.88 | | | $7.12 | | $21.87 | | $2.83 | | $2.55 | |
| Physiotherapist | 0.002 | 0.002 | $30.36 | $0.07 | | $0.07 | | $64.50 | | $0.15 | | | $0.15 | | $27.76 | | $0.06 | | $0.06 | |
| CT scan | 0.500 | 1.000 | $178.55 | $89.28 | | $178.55 | | $1,463.00 | | $731.50 | | | $1,463.00 | | $210.08 | | $105.04 | | $210.08 | |
| MRI | 0.500 | 1.000 | $368.12 | $184.06 | | $368.12 | | $3,445.00 | | $1,722.50 | | | $3,445.00 | | $535.26 | | $267.63 | | $535.26 | |
| Respiratory surgical procedure | 0.008 | 0.008 | $28,414.94 | $221.98 | | $221.98 | | $27,562.88 | | $215.32 | | | $215.32 | | $35,438.16 | | $276.84 | | $276.84 | |
| Ultrasound | 0.500 | 1.000 | $108.97 | $54.49 | | $108.97 | | $330.50 | | $165.25 | | | $330.50 | | $64.70 | | $32.35 | | $64.70 | |
| X-ray | 0.500 | 1.000 | $80.90 | $40.45 | | $80.90 | | $130.50 | | $65.25 | | | $130.50 | | $32.73 | | $16.37 | | $32.73 | |
| Radiotherapy | 0.003 | 0.003 | $120.54 | $0.37 | | $0.37 | | $325.00 | | $0.99 | | | $0.99 | | $134.69 | | $0.41 | | $0.41 | |
| Unscheduled hospitalization day | 0.072 | 1.717 | $2,394.45 | $171.47 | | $4,110.48 | | $2,557.24 | | $183.12 | | | $4,389.93 | | $2,551.89 | | $182.74 | | $4,380.74 | |
| ICU visit | 0.034 | 0.133 | *Included in hospital stay cost* | $0.00 | | $0.00 | | *Included in hospital stay cost* | | $0.00 | | | $0.00 | | *Included in hospital stay cost* | | $0.00 | | $0.00 | |
| ED visit | 0.055 | 0.017 | $725.96 | $40.22 | | $12.10 | | $615.97 | | $34.13 | | | $10.27 | | $461.97 | | $25.60 | | $7.70 | |
| Home care services | 0.000 | 0.567 | $129.10 | $0.00 | | $73.16 | | $241.50 | | $0.00 | | | $136.85 | | $116.06 | | $0.00 | | $65.77 | |
| **Total** |  |  |  | **$873.63** | | **$5,462.06** | |  | | **$3,238.45** | | | **$10,658.77** | |  | | **$972.45** | | **$5,857.96** | |
| **Source** | **Graham et al. (2016),**^2^ **Dalal. et al. (2018),**^3^ **and KOL assumption** | | **CMS.gov**^4,5^ | | | | | | **InHealth Professional Services**^6^ | | | | | | | **DC.gov**^7^ | | | | |

CT, computed tomography; ED, emergency department; GP, general practitioner; ICU, intensive care unit; KOL, key opinion leader; MRI, magnetic resonance imaging.

## Appendix 3. Adverse Event Cost Calculations

**Table 3.** Adverse Event Cost Calculations

| **Regimens** | **Medicare cost per event**^4,8^***** | **Commercial cost per event**^8,9^***** | **Medicaid cost per event**^7,8^ | **Tepotinib**^10^ | **Capmatinib**^11^ |
| --- | --- | --- | --- | --- | --- |
| Diarrhea | $10,037 | $9,418 | $10,953 | 0.4% | 0.3% |
| Dyspnea | $7,182 | $7,752 | $7,457 | 2.0% | 7.0% |
| Fatigue | $9,649 | $9,508 | $9,401 | 1.6% | 8.0% |
| Nausea | $10,037 | $9,418 | $10,953 | 0.8% | 2.7% |
| Peripheral edema | $9,649 | $9,508 | $9,401 | 9.0% | 9.0% |
| Pleural effusion | $11,162 | $11,347 | $12,166 | 5.0% | – |
| Pneumonitis/pneumonia | $13,503 | $15,480 | $18,368 | 3.9% | – |
| Vomiting | $10,037 | $9,418 | $10,953 | 1.2% | 2.4% |
| Medicare | *N/A* | *N/A* | *N/A* | $2,492 | $2,685 |
| Commercial | *N/A* | *N/A* | *N/A* | $2,560 | $2,668 |
| Medicaid | *N/A* | *N/A* | *N/A* | $2,733 | $2,712 |

*Rounded to the nearest US Dollar.

N/A, not applicable.

## Appendix 4. Subsequent Treatment Cost Calculations

Assumptions regarding subsequent therapies were as follows:

- It was assumed 30.1% of patients would receive subsequent treatment after discontinuing 2L+,^10^ including 8.4% of patients who would receive a tyrosine kinase inhibitor (TKI), and 10.8%, 3.0%, 3.6%, and 4.2% who would receive immune checkpoint inhibitor (ICI) monotherapy, ICI + chemotherapy (with or without anti-vascular endothelial growth factor [VEGF]), anti-VEGF (with or without chemotherapy), and chemotherapy alone, respectively
- Patients who had already received a TKI could receive another TKI, but not the same product. 1.8% of patients who had received tepotinib would receive capmatinib, while 6.6% received crizotinib; by contrast, 8.4% of patients who had received capmatinib were subsequently administered crizotinib
- Subsequent treatment costs were applied for a period of 3 months (based on mean PFS for subsequent treatments in the VISION trial)^10^

Subsequent treatment expenses included drug acquisition and administration (see table below):

- From the Medicare health plan perspective, the cost of IV infusion was $311 for the first hour, $62 for any additional hours, and $62 for subsequent therapies requiring infusion.^5^ Subsequent treatment costs following discontinuation from tepotinib and capmatinib were therefore calculated to be $14,428 and $14,335, respectively
- From the commercial health plan perspective, the cost of IV infusion was $514 for the first hour, $109 for any additional hours, and $246 for subsequent therapies requiring infusion.^9^ Subsequent treatment costs following discontinuation from tepotinib and capmatinib were therefore calculated to be $14,644 and $14,550, respectively
- From the Medicaid health plan perspective, the cost of IV infusion was $145 for the first hour, $30 for any additional hours, and $70 for subsequent therapies requiring infusion.^7^ Subsequent treatment costs following discontinuation from tepotinib and capmatinib were therefore calculated to be $14,323 and $14,230, respectively

**Table 4.** Drug Acquisition and Administration Costs for Subsequent Therapy (Medicare Health Plan Perspective)

| **Intervention** | **Regimen** | **Fixed Drug Acquisition** | **Monthly Drug Acquisition** | **Fixed Drug Admin.** | **Monthly Drug Admin.** | **Source, Assumptions** |
| --- | --- | --- | --- | --- | --- | --- |
| Tepotinib | Tepotinib | $0.00 | $21,130.83 | $0.00 | $0 | **Cost:** Manufacturer provided^10^  **Dosing:** TEPMETKO PI^12^ |
| Capmatinib | Capmatinib | $0.00 | $20,515.39 | $0.00 | $0 | **Cost:** Micromedex Redbook^®13^  **Dosing:** TABRECTA PI^11^ |
| Crizotinib | Crizotinib | $0.00 | $18,793.15 | $0.00 | $0 | **Cost:** Micromedex Redbook^®13^  **Dosing:** XALKORI PI^14^ |
| Experimental treatment* | Undefined | $0.00 | $0.00 | $0.00 | $0 | All values assumed equal to crizotinib |
| ICI monotherapy | Atezolizumab | $0.00 | $13,678.67 | $0.00 | $448.86 | **Cost:** Micromedex Redbook^®13^  **Dosing:** TECENTRIQ PI^15^; OPDIVO PI^16^; KEYTRUDA PI^17^ |
|  | Nivolumab | $0.00 | $14,688.57 | $0.00 | $336.65 |  |
|  | Pembrolizumab | $0.00 | $14,541.74 | $0.00 | $448.86 |  |
| ICI + chemotherapy ± anti-VEGF | Pembrolizumab  Carboplatin  Pemetrexed | $0.00  $237.28  $0.00 | $14,541.74  $0.00  $10,005.58 | $0.00 | $448.86  0  $89.51 | **Cost:** Micromedex Redbook^®13^  **Dosing:** KEYTRUDA PI^17^; Socinski et al. (2018)^18^ |
|  |  |  |  | $247.88 |  |  |
|  |  |  |  | $0.00 |  |  |
|  | Atezolizumab  Carboplatin  Paclitaxel  Bevacizumab | $0.00  $296.60  $341.50  $0.00 | $13,678.67  $0.00  $0.00  $11,511.36 | $0.00 | $448.86  $0  $0  $179.02 |  |
|  |  |  |  | $309.85 |  |  |
|  |  |  |  | $619.70 |  |  |
|  |  |  |  | $0.00 |  |  |
| Chemotherapy + anti-VEGF | Paclitaxel  Carboplatin  Bevacizumab | $409.80  $355.92  $0.00 | $0.00  $0.00  $11,511.36 | $743.64 | $0  $0  $538.37 | **Cost:** Micromedex Redbook^®13^  **Dosing:** Sandler et al. (2006)^19^; Rodriguez-Abreu et al. (2021)^20^; Barlesi et al. (2013)^21^; Shimizu et al. (2019)^22^; Stefanou et al. (2016)^23^ |
|  |  |  |  | $371.82 |  |  |
|  |  |  |  | $0.00 |  |  |
|  | Pemetrexed  Bevacizumab | $0.00  $0.00 | $10,005.58  $5,755.68 | $0.00 | $89.51  $538.37 |  |
|  |  |  |  | $0.00 |  |  |
|  | Docetaxel  Bevacizumab | $0.00  $0.00 | $221.00  $11,511.36 | $0.00 | $89.51  $448.86 |  |
|  |  |  |  | $0.00 |  |  |
|  | Pemetrexed  Carboplatin Bevacizumab | $0.00  $355.92  $0.00 | $10,005.58  0.00  $11,511.36 | $0.00 | $89.51  $0  $538.37 |  |
|  |  |  |  | $371.82 |  |  |
|  |  |  |  | $0.00 |  |  |
| Chemotherapy alone | Docetaxel | $0.00 | $221.00 | $0.00 | $448.86 | **Cost:** Micromedex Redbook^®13^  **Dosing:** Docetaxel PI^24^; ALMITA PI^25^; Gervais et al. (2013)^26^; Sandler et al. (2006)^19^ |
|  | Pemetrexed | $0.00 | $10,005.58 | $0.00 | $448.86 |  |
|  | Carboplatin  Pemetrexed | $355.92  $0.00 | $0.00  $10,005.58 | $1,864.50 | $0  $89.51 |  |
|  |  |  |  | $0.00 |  |  |
|  | Cisplatin  Pemetrexed | $261.18  $0.00 | $0.00  $10,005.58 | $2,236.32 | $0  $89.51 |  |
|  |  |  |  | $0.00 |  |  |
|  | Carboplatin  Paclitaxel | $355.92  $409.80 | $0.00  $0.00 | $1,864.50 | $0  $0 |  |
|  |  |  |  | $743.64 |  |  |

*The model assumes that payers will not bear the cost of drugs used in clinical trials.

ICI, immune checkpoint inhibitor; VEGF, vascular endothelial growth factor; PI, prescribing information.

## Appendix 5. Treatment Monitoring Cost Calculations

The cost-effectiveness model also incorporated the cost of treatment monitoring. From the Medicare perspective, the unit cost of testing related to hematology and coagulation (i.e., blood test), biochemistry (or liver function tests), electrolytes, and urine analysis was $6.47, $8.46, $7.01, and $3.17, respectively.^5^ As per KOL assumption, it was assumed patients would receive one of each type of test per month, irrespective of the treatment they were receiving, implying monthly monitoring costs of $25.11 per patient.

The scenario analyses applied alternative costs for commercial health plan and Medicaid health plan perspectives, although utilization was assumed similar to Medicare health plan:

- From the commercial health plan perspective, the unit cost of testing related to hematology and coagulation, biochemistry, electrolytes, and urine analysis was $44.00, $57.00, $52.00, and $35.50, respectively.^9^ This implied monthly monitoring costs of $188.50
- From a Medicaid health plan perspective, the unit cost of testing related to hematology and coagulation, biochemistry, electrolytes, and urine analysis was $5.18, $6.77, $5.61, and $2.54, respectively.^5^ This implied monthly monitoring costs of $20.10

## Appendix 6. Derivation of Health-related Quality-of-life Inputs

In accordance with current health technology assessment guidelines adopted by the National Institute for Health and Care Excellence (NICE),^27^ the analysis cross-walked EQ-5D-5L trial data to EQ-5D-3L using the mapping algorithm developed by van Hout et al. (2012);^28^ the resultant EQ-5D-3L scores were then converted to index values using country-specific value sets.^29^ Finally, linear mixed models were fitted to account for changes in utility attributable to progression status (no difference was observed between treatment lines). No published utility values were available for capmatinib, however as tepotinib and capmatinib represent the same class of products and share a similar mode of administration, and since the CEM explicitly captures differences in safety profiles that may yield differences in health-related quality-of-life (HRQoL), utility weights derived from the US statistical model were applied to each drug in the model.

Derivation of utility decrements associated with incidence of AEs in the CEM is summarized in the table below.

**Table 5.** Derivation of AE-Related Utility Decrements

| **Adverse Events** | **Disutilities** | **Duration (Days)** | **Disutilities Decrement** | **Source disutility** |
| --- | --- | --- | --- | --- |
| Diarrhea | -0.0470 | 30.42 | -0.0039 | ICER^30^ |
| Dyspnea | -0.0500 | 30.42 | -0.0042 | ICER^30^ |
| Fatigue | -0.0735 | 30.42 | -0.0061 | ICER^30^ |
| Nausea | -0.0480 | 30.42 | -0.0040 | ICER^30^ |
| Peripheral edema | -0.0615 | 30.42 | -0.0051 | Assumption: average of other AE disutility, confirmed by KOLs |
| Pleural effusion | 0.0000 | 30.42 | 0.0000 | Assumption: zero as in NICE^31^ |
| Pneumonitis/pneumonia | -0.0730 | 30.42 | -0.0061 | ICER^30^ |
| Vomiting | -0.0615 | 30.42 | -0.0051 | Assumption: average of other AE disutility, confirmed by KOLs |

AE, adverse event; ICER, Institute for Clinical and Economic Review; KO, key opinion leader; NICE, National Institute for Health and Care Excellence; resp, respiratory.

## Appendix 7. Summary of Model Scenarios

**Table 6.** Scenario Rationale and Design

| **Scenario** | **Description** | **Rationale** |
| --- | --- | --- |
| Assume treat until progression | This scenario assumes patients always (and only) discontinue treatment upon disease progression. This is operationalized in the model by using the PFS curves for tepotinib (extrapolated from VISION through parametric survival analysis) and capmatinib (derived from the tepotinib PFS curve by applying the HR derived from the unanchored MAIC) in lieu of the predicted TTD curve and median duration of exposure, respectively. | While the base-case analysis was designed to account for varying strategies around discontinuation (e.g., for the tendency for some patients to discontinue treatment prior to progression, whereas others may continue until or past progression), this scenario sought to examine how cost-effectiveness results might vary in contexts where termination of therapy takes place primarily when a patient’s cancer progresses. This was seen as particularly important given large observed differences in median PFS and TTD between the model comparators (see Table 1). |
| Include biomarker testing costs | This scenario applies a one-off cost for biomarker testing of $2,854.73, which was calculated as an average over six tests presently available in the US (i.e., Foundation Medicine Inc., Guardant Health, Oncomine Solutions, Caris, Neogenomics, and ArcherDx) using data derived from the Centers for Medicare and Medicaid Services.^32^ | Incorporating biomarker testing costs is not expected to impact the model results (since it applies equally to both comparators) but was thought to be of potential interest for its ability to quantify the relative contribution of testing to overall treatment costs associated with this indication. |
| Employ alternative DM resource utilization | Applies the following values for selected categories of resource utilization (all monthly) from the original publications by Graham 2016 (PF) and Dalal 2018 (PD):^2,3^   - Specialist visit: 0.147 for PF; 0.15 for PD - CT scan: 0.033 in PF/PD - MRI: 0.01 in PF/PD - Ultrasound: 0.008 in PF/PD - X-Ray: 0.041 in PF/PD | US KOLs believed values for imaging and specialist visits for PF and PD were low, and it should be approximately bi-monthly in PF and monthly in PD (as used in the base-case). This scenario assesses the implications of applying the original published values. |
| Exclude subsequent treatment expenditures | Excludes costs associated with subsequent treatment from analytical results. | KOLs expressed concerns that the frequency of subsequent therapy observed in VISION (30.1%) may not accurately represent real-world clinical practice. Accordingly, these scenarios examined the implications of uncertainty in this aspect of the model. |
| Double subsequent treatment frequencies | Assumes 60.3% of patients receive subsequent therapy following disease progression (versus 30.1% in the base-case). This is achieved by doubling the proportion of patients who are administered each treatment across all patient populations (i.e., such that each treatment’s share remains constant). |  |
| Literature-based PF and PD utility values | Applies utilities for progression-free (0.65) and progressed (0.47) disease drawn from a report published by the Institute for Clinical and Economic Review.^30^ | KOLs requested to explore alternative utility values for the progressed state, as they perceived the value derived from VISION may be too high. |
| Exclude AE disutilities | This scenario assumes experiencing AEs does not significantly impact health-related quality of life (i.e., all utility decrements are set to 0). Costs associated with AEs are retained. | This scenario was included to explore the significance of differences in safety profiles between tepotinib and capmatinib (summarized in Appendix 3 above) for model results. |
| 5-year time horizon | The model records costs and utilities for the patient cohort over the first five years only. | These scenarios probed the impact of constraining or expanding the model horizon. A 20-year time horizon was considered given that a similar horizon had been applied in several prior health economic evaluations for advanced NSCLC.^33–35^ |
| 20-year time horizon | The model records costs and utilities for the patient cohort over 20 years. |  |
| 5% cost and health outcomes discount rates | As described in the Scenario column. | Current health economic guidelines for the US advise that while a 3% real discount rate is most appropriate for the purposes of conducting CEA, a range of rates should be considered in sensitivity analyses.^36^ |
| 0% cost and health outcomes discount rates | As described in the Scenario column. |  |
| Commercial perspective | In this scenario, drug administration costs (including those incorporated into subsequent therapy) and expenditures associated with treatment monitoring and AE and disease management reflect the commercial context.^6^ | It is important to assess the cost-effectiveness of tepotinib outside the Medicare context in the event it is reimbursed by commercial and Medicare payers. |
| Medicaid perspective | In this scenario, drug administration costs (including those incorporated into subsequent therapy) and expenditures associated with treatment monitoring and AE and disease management reflect the Medicaid context.^7^ |  |
| Apply population weighting from Flatiron | This scenario assumed 1L patients would compromise 56.4% of recipients of tepotinib/capmatinib, while the remaining 44.4% would receive it in 2L+ (by contrast, in VISION, 44.5% and 55.5% of trial participants were 1L and 2L+, respectively).^37,38^ | This scenario was intended to evaluate the impact of uncertainty regarding utilization of tepotinib and capmatinib in clinical practice and was considered of especial importance given divergence in base-case results between the 1L and 2L+ patient populations. |
| Apply $35 co-payment | This scenario assumed a $35 co-payment applies to the per-package cost of tepotinib and capmatinib. | These scenarios were intended to explore the impact of patient cost-sharing on model results. The specific values employed in the analysis were arbitrarily selected. |
| Apply 10% co-insurance | This scenario assumed patients bear 10% of drug acquisition costs associated with tepotinib and capmatinib. |  |
| Alternative distributions for tepotinib OS curves | As described in the Scenario column. Alternative distributions applied in the scenario analyses include Weibull, lognormal, log-logistic, gamma, and Gompertz. | Although the parametric survival analysis used to extrapolate OS, PFS, and TTD beyond the VISION trial follow-up period were conducted in accordance with current best practice and supported by KOL opinion (exponential), these extrapolations invariably introduce uncertainty into the analysis. Accordingly, alternative distributions were tested for all patient populations to assess the impact on model results. |
| Alternative distributions for tepotinib PFS curves |  |  |
| Alternative distributions for tepotinib TTD curves |  |  |

**Table 7.** Supplementary Scenario Analysis Results

| **Scenario** | **1L** | | | | | | **2L+** | | | | | | **Line Agnostic (1L & 2L+)** | | | | | |
| --- | --- | --- | --- | --- | --- | --- | --- | --- | --- | --- | --- | --- | --- | --- | --- | --- | --- | --- |
|  | **Costs ($)** | | | **QALYs** | | | **Costs ($)** | | | **QALYs** | | | **Costs ($)** | | | **QALYs** | | |
|  | **TEP** | **CAP** | **Incr.** | **TEP** | **CAP** | **Incr.** | **TEP** | **CAP** | **Incr.** | **TEP** | **CAP** | **Incr.** | **TEP** | **CAP** | **Incr.** | **TEP** | **CAP** | **Incr.** |
| **Base-case** | **347,719** | **395,475** | **-47,756** | **1.4363** | **1.2236** | **0.2127** | **342,615** | **313,168** | **91,401** | **1.4121** | **1.0791** | **0.3330** | **342,615** | **313,168** | **29,447** | **1.4229** | **1.1435** | **0.2794** |
| Assume treat until progression | 426,326 | 358,811 | 67,515 | 1.4363 | 1.2236 | 0.2127 | 371,196 | 255,258 | 115,938 | 1.4121 | 1.0791 | 0.3330 | 395,740 | 301,360 | 94,380 | 1.4229 | 1.1435 | 0.2794 |
| Include biomarker testing costs | 350,573 | 398,330 | -47,756 | 1.4363 | 1.2236 | 0.2127 | 341,375 | 249,974 | 91,401 | 1.4121 | 1.0791 | 0.3330 | 345,470 | 316,023 | 29,447 | 1.4229 | 1.1435 | 0.2794 |
| Employ alternative DM resource utilization | 334,058 | 383,857 | -49,799 | 1.4363 | 1.2236 | 0.2127 | 323,333 | 234,411 | 88,922 | 1.4121 | 1.0791 | 0.3330 | 328,108 | 300,945 | 27,163 | 1.4229 | 1.1435 | 1.4229 |
| Exclude subsequent treatment expenditures | 342,453 | 390,312 | -47,859 | 1.4363 | 1.2236 | 0.2127 | 331,148 | 238,082 | 93,066 | 1.4121 | 1.0791 | 0.3330 | 336,181 | 305,856 | 30,325 | 1.4229 | 1.1435 | 1.4229 |
| Double subsequent treatment frequencies | 352,984 | 400,638 | -47,654 | 1.4363 | 1.2236 | 0.2127 | 345,892 | 256,156 | 89,736 | 1.4121 | 1.0791 | 0.3330 | 349,050 | 320,480 | 28,569 | 1.4229 | 1.1435 | 1.4229 |
| Literature-based PF and PD utility values | 347,719 | 395,475 | -47,756 | 1.2187 | 1.0385 | 0.1801 | 338,520 | 247,119 | 91,401 | 1.1675 | 0.8730 | 0.2945 | 342,615 | 313,168 | 29,447 | 1.1902 | 0.9467 | 1.1902 |
| Exclude AE disutilities | 347,719 | 395,475 | -47,756 | 1.4373 | 1.2251 | 0.2122 | 338,520 | 247,119 | 91,401 | 1.4131 | 1.0806 | 0.3325 | 342,615 | 313,168 | 29,447 | 1.4239 | 1.1449 | 1.4239 |
| 5-year time horizon | 335,921 | 383,433 | -47,512 | 1.3258 | 1.1610 | 0.1648 | 326,479 | 242,504 | 83,975 | 1.3032 | 1.0363 | 0.2668 | 330,683 | 305,246 | 25,436 | 1.3132 | 1.0918 | 0.2214 |
| 20-year time horizon | 348,808 | 395,996 | -47,188 | 1.4469 | 1.2274 | 0.2195 | 339,639 | 247,328 | 92,311 | 1.4226 | 1.0811 | 0.3415 | 343,721 | 313,516 | 30,205 | 1.4334 | 1.1462 | 0.2872 |
| 5% cost and health outcomes discount rates | 342,648 | 388,596 | -45,948 | 1.4054 | 1.1988 | 0.2067 | 333,678 | 244,389 | 89,289 | 1.3820 | 1.0589 | 0.3231 | 337,671 | 308,591 | 29,081 | 1.3924 | 1.1211 | 0.2713 |
| 0% cost and health outcomes discount rates | 359,120 | 408,265 | -49,145 | 1.5174 | 1.2754 | 0.2420 | 349,638 | 252,184 | 97,454 | 1.4918 | 1.1181 | 0.3737 | 353,860 | 321,672 | 32,188 | 1.5032 | 1.1881 | 0.3151 |
| Commercial perspective | 435,901 | 470,325 | -34,424 | 1.4469 | 1.2274 | 0.2195 | 436,552 | 327,725 | 108,827 | 1.4226 | 1.0811 | 0.3415 | 436,262 | 391,212 | 45,051 | 1.4334 | 1.1462 | 0.2872 |
| Medicaid perspective | 354,102 | 400,246 | -46,144 | 1.4469 | 1.2274 | 0.2195 | 345,970 | 252,650 | 93,320 | 1.4226 | 1.0811 | 0.3415 | 349,590 | 318,361 | 31,230 | 1.4334 | 1.1462 | 0.2872 |
| Apply population weighting from Flatiron | 347,719 | 395,475 | -47,756 | 1.4363 | 1.2236 | 0.2127 | 338,520 | 247,119 | 91,401 | 1.4121 | 1.0791 | 0.3330 | 343,644 | 329,753 | 13,890 | 1.4256 | 1.1596 | 0.2660 |
| Apply $35 co-payment | 347,203 | 394,198 | -46,995 | 1.4363 | 1.2236 | 0.2127 | 338,027 | 246,428 | 91,598 | 1.4121 | 1.0791 | 0.3330 | 342,112 | 312,216 | 29,896 | 1.4229 | 1.1435 | 0.2794 |
| Apply 10% co-insurance | 319,881 | 361,953 | -42,072 | 1.4363 | 1.2236 | 0.2127 | 313,227 | 230,229 | 82,998 | 1.4121 | 1.0791 | 0.3330 | 316,189 | 288,873 | 27,316 | 1.4229 | 1.1435 | 0.2794 |
| Weibull distribution for tepotinib OS curve | 342,303 | 391,869 | -49,567 | 1.3851 | 1.1889 | 0.1962 | 324,189 | 241,311 | 82,878 | 1.2726 | 1.0146 | 0.2580 | 332,253 | 308,341 | 23,913 | 1.3227 | 1.0922 | 0.2305 |
| Lognormal distribution for tepotinib OS curve | 376,831 | 417,789 | -40,958 | 1.7123 | 1.4348 | 0.2775 | 349,858 | 254,843 | 95,015 | 1.5174 | 1.1461 | 0.3713 | 361,866 | 327,387 | 34,479 | 1.6042 | 1.2746 | 0.3295 |
| Log-logistic distribution for tepotinib OS curve | 369,759 | 411,935 | -42,176 | 1.6456 | 1.3794 | 0.2662 | 349,836 | 255,033 | 94,803 | 1.5172 | 1.1472 | 0.3700 | 358,706 | 324,887 | 33,819 | 1.5744 | 1.2506 | 0.3238 |
| Gamma distribution for tepotinib OS curve | 356,101 | 401,584 | -45,483 | 1.5146 | 1.2800 | 0.2346 | 340,354 | 249,290 | 91,063 | 1.4260 | 1.0918 | 0.3342 | 347,364 | 317,092 | 30,272 | 1.4654 | 1.1756 | 0.2898 |
| Gompertz distribution for tepotinib OS curve | 348,412 | 395,948 | -47,535 | 1.4429 | 1.2281 | 0.2148 | 322,635 | 239,947 | 82,688 | 1.2599 | 1.0059 | 0.2540 | 334,111 | 309,400 | 24,712 | 1.3414 | 1.1048 | 0.2366 |
| Weibull distribution for tepotinib PFS curve | 347,432 | 395,308 | -47,877 | 1.4368 | 1.2238 | 0.2129 | 340,048 | 246,125 | 93,923 | 1.4098 | 1.0802 | 0.3295 | 343,335 | 312,542 | 30,793 | 1.4218 | 1.1442 | 0.2776 |
| Lognormal distribution for tepotinib PFS curve | 333,700 | 386,280 | -52,581 | 1.4566 | 1.2367 | 0.2199 | 332,501 | 245,865 | 86,636 | 1.4202 | 1.0801 | 0.3401 | 333,035 | 308,378 | 24,656 | 1.4364 | 1.1498 | 0.2866 |
| Log-logistic distribution for tepotinib PFS curve | 336,602 | 388,827 | -52,224 | 1.4526 | 1.2332 | 0.2194 | 329,488 | 245,301 | 84,187 | 1.4248 | 1.0810 | 0.3439 | 332,656 | 309,199 | 23,456 | 1.4372 | 1.1488 | 0.2884 |
| Gamma distribution for tepotinib PFS curve | 333,082 | 385,816 | -52,734 | 1.4575 | 1.2374 | 0.2202 | 328,832 | 245,332 | 83,499 | 1.4257 | 1.0808 | 0.3448 | 330,724 | 307,877 | 22,847 | 1.4398 | 1.1505 | 0.2893 |
| Gompertz distribution for tepotinib PFS curve | 330,743 | 384,233 | -53,490 | 1.4615 | 1.2402 | 0.2212 | 335,192 | 247,331 | 87,860 | 1.4168 | 1.0789 | 0.3379 | 333,211 | 308,281 | 24,930 | 1.4367 | 1.1507 | 0.2860 |

AE, adverse event; ICER, Institute for Clinical and Economic Review; KO, key opinion leader; NICE, National Institute for Health and Care Excellence; resp, respiratory.

## Appendix 8. Summary of Deterministic Sensitivity Analysis Results from the Medicare Health Plan Perspective: Incremental Net Monetary Benefit (INMB)

**Table 8.** Results for 1L Patient Population

| **Rank** | **Parameter** | **Lower Bound** | **Upper Bound** | **Absolute Difference** |
| --- | --- | --- | --- | --- |
| 1 | Tepotinib TTD: Exponential Distribution Parameter | $5,554 | $137,953 | $132,398 |
| 2 | Capmatinib Median Treatment Duration | $17,611 | $140,498 | $122,887 |
| 3 | Drug acquisition, Cost per month: Capmatinib | $30,144 | $129,188 | $99,044 |
| 4 | Capmatinib PFS: HR | $31,768 | $130,596 | $98,828 |
| 5 | Drug acquisition, Cost per month: Tepotinib | $120,646 | $38,686 | $81,959 |
| 6 | Capmatinib OS: HR | $34,886 | $7,875 | $27,011 |
| 7 | Tepotinib PFS: Exponential Distribution Parameter | $83,541 | $76,497 | $7,044 |
| 8 | Tepotinib OS: Exponential Distribution Parameter | $81,788 | $77,542 | $4,246 |
| 9 | Disease management, Post-progression | $80,773 | $78,559 | $2,214 |
| 10 | Utilities: Pre-progression | $78,703 | $80,629 | $1,926 |
| 11 | SubTx One-off Cost: Tepotinib | $80,456 | $78,876 | $1,580 |
| 12 | SubTx One-off Cost: Capmatinib | $78,892 | $80,440 | $1,549 |
| 13 | Utilities: Post-progression | $79,206 | $80,126 | $920 |
| 14 | AE Management, One-off Cost: Capmatinib | $79,263 | $80,069 | $806 |
| 15 | AE Management, One-off Cost: Tepotinib | $80,040 | $79,292 | $748 |
| 16 | Disease management, Pre-progression | $79,973 | $79,359 | $613 |
| 17 | Treatment monitoring, Total cost per month: Capmatinib | $79,607 | $79,725 | $118 |
| 18 | Treatment monitoring, Total cost per month: Tepotinib | $79,713 | $79,619 | $94 |
| 19 | One-off Disutilities: Capmatinib | $79,633 | $79,699 | $67 |
| 20 | One-off Disutilities: Tepotinib | $79,688 | $79,644 | $44 |
| 21 | Disease management, One-off Cost: EOL | $79,656 | $79,676 | $21 |
| 22 | Disease management, One-off Cost: Progression | $79,667 | $79,665 | $2 |

1L, first line; 2L+, second line or later; OS, overall survival; PFS, progression-free survival; TTD, time to discontinuation; EOL, end-of-life; HR, hazard ratio; AE, adverse event; SubTx, subsequent treatment.

**Table 9.** Results for 2L+ Patient Population

| **Rank** | **Parameter** | **Lower Bound** | **Upper Bound** | **Absolute Difference** |
| --- | --- | --- | --- | --- |
| 1 | Tepotinib TTD: Exponential Distribution Parameter | -$99,238 | $5,207 | $104,445 |
| 2 | Drug acquisition, Cost per month: Tepotinib | -$4,606 | -$78,309 | $73,704 |
| 3 | Capmatinib Median Treatment Duration | -$71,002 | -$12,100 | $58,902 |
| 4 | Capmatinib PFS: HR | -$72,439 | -$22,085 | $50,355 |
| 5 | Drug acquisition, Cost per month: Capmatinib | -$65,459 | -$17,456 | $48,003 |
| 6 | Capmatinib OS: HR | -$68,246 | -$26,710 | $41,536 |
| 7 | Tepotinib PFS: Exponential Distribution Parameter | -$33,672 | -$47,703 | $14,031 |
| 8 | Tepotinib OS: Exponential Distribution Parameter | -$38,797 | -$43,915 | $5,118 |
| 9 | Utilities: Pre-progression | -$43,508 | -$39,408 | $4,100 |
| 10 | Sub Tx One-off Cost: Capmatinib | -$42,813 | -$40,102 | $2,711 |
| 11 | Sub Tx One-off Cost: Tepotinib | -$40,352 | -$42,563 | $2,212 |
| 12 | Disease management, Pre-progression | -$40,805 | -$42,110 | $1,305 |
| 13 | Disease management, Post-progression | -$40,951 | -$41,964 | $1,013 |
| 14 | AE Management, One-off Cost: Capmatinib | -$41,860 | -$41,055 | $806 |
| 15 | AE Management, One-off Cost: Tepotinib | -$41,084 | -$41,831 | $748 |
| 16 | Utilities: Post-progression | -$41,668 | -$41,247 | $421 |
| 17 | Treatment monitoring, Total cost per month: Tepotinib | -$41,416 | -$41,500 | $84 |
| 18 | One-off Disutilities: Capmatinib | -$41,491 | -$41,424 | $67 |
| 19 | Treatment monitoring, Total cost per month: Capmatinib | -$41,485 | -$41,430 | $55 |
| 20 | One-off Disutilities: Tepotinib | -$41,435 | -$41,480 | $44 |
| 21 | Disease management, One-off Cost: Progression | -$41,477 | -$41,438 | $39 |
| 22 | Disease management, One-off Cost: EOL | -$41,473 | -$41,442 | $31 |

1L, first line; 2L+, second line or later; AE, adverse event; EOL, end-of-life; HR, hazard ratio; OS, overall survival; PFS, progression-free survival; SubTx, subsequent treatment; TTD, time to discontinuation.

**Table 10.** Results for Line Agnostic (1L & 2L+) Patient Population

| **Rank** | **Parameter** | **Lower Bound** | **Upper Bound** | **Absolute Difference** |
| --- | --- | --- | --- | --- |
| 1 | Drug acquisition, Cost per month: Tepotinib | $51,157 | -$26,222 | $77,379 |
| 2 | Drug acquisition, Cost per month: Capmatinib | -$22,896 | $47,831 | $70,727 |
| 3 | Tepotinib TTD: Exponential Distribution Parameter (1L) | -$20,528 | $38,417 | $58,944 |
| 4 | Tepotinib TTD: Exponential Distribution Parameter (2L+) | -$19,589 | $38,356 | $57,946 |
| 5 | Capmatinib Median Treatment Duration (1L) | -$15,160 | $39,550 | $54,710 |
| 6 | Capmatinib PFS: HR (1L) | -$8,857 | $35,141 | $43,999 |
| 7 | Capmatinib Median Treatment Duration (2L+) | -$3,924 | $28,755 | $32,679 |
| 8 | Capmatinib PFS: HR (2L+) | -$4,721 | $23,215 | $27,937 |
| 9 | Capmatinib OS: HR (2L+) | -$2,395 | $20,649 | $23,044 |
| 10 | Capmatinib OS: HR (1L) | -$7,469 | -$19,494 | $12,025 |
| 11 | Tepotinib PFS: Exponential Distribution Parameter (2L+) | $16,787 | $9,002 | $7,784 |
| 12 | Tepotinib PFS: Exponential Distribution Parameter (1L) | $14,192 | $11,056 | $3,136 |
| 13 | Utilities: Pre-progression | $10,901 | $14,033 | $3,132 |
| 14 | Tepotinib OS: Exponential Distribution Parameter (2L+) | $13,943 | $11,104 | $2,839 |
| 15 | Tepotinib OS: Exponential Distribution Parameter (1L) | $13,412 | $11,522 | $1,890 |
| 16 | Disease management, Post-progression | $13,241 | $11,693 | $1,548 |
| 17 | Sub Tx One-off Cost: Capmatinib (2L+) | $11,715 | $13,219 | $1,504 |
| 18 | Sub Tx One-off Cost: Tepotinib (2L+) | $13,081 | $11,854 | $1,227 |
| 19 | Disease management, Pre-progression | $12,966 | $11,969 | $997 |
| 20 | AE Management, One-off Cost: Capmatinib | $12,065 | $12,870 | $806 |
| 21 | AE Management, One-off Cost: Tepotinib | $12,841 | $12,093 | $748 |
| 22 | SubTx One-off Cost: Tepotinib (1L only) | $12,819 | $12,116 | $703 |
| 23 | SubTx One-off Cost: Capmatinib (1L only) | $12,123 | $12,812 | $690 |
| 24 | Utilities: Post-progression | $12,146 | $12,789 | $643 |
| 25 | Treatment monitoring, Total cost per month: Tepotinib | $12,511 | $12,423 | $88 |
| 26 | Treatment monitoring, Total cost per month: Capmatinib | $12,426 | $12,509 | $83 |
| 27 | One-off Disutilities: Capmatinib | $12,434 | $12,501 | $67 |
| 28 | One-off Disutilities: Tepotinib | $12,490 | $12,445 | $44 |
| 29 | Disease management, One-off Cost: EOL | $12,454 | $12,481 | $26 |
| 30 | Disease management, One-off Cost: Progression | $12,457 | $12,478 | $21 |

1L, first line; 2L+, second line or later; AE, adverse event; EOL, end-of-life; HR, hazard ratio; OS, overall survival; PFS, progression-free survival; SubTx, subsequent treatment; TTD, time to discontinuation.

## References

1. The healthcare business of Merck KGaA, Darmstadt, Germany. Tepotinib vs. Comparator, KM & survival parameters. Data on file. 2021.

2. Graham, J., Earnshaw, S., Lim, J., Luthra, R., & Borker R. Cost-effectiveness of Afatinib versus Erlotinib in the First-line Treatment of Patients with Metastatic Non-small Cell Lung Cancer with EGFR Exon 19 Deletion Mutations. *J Clin Pathways*. 2016;2(4):31-39.

3. Dalal AA, Guerin A, Mutebi A, Culver KW. Treatment patterns, clinical and economic outcomes of patients with anaplastic lymphoma kinase-positive non-small cell lung cancer receiving ceritinib: a retrospective observational claims analysis. *J Drug Assess*. 2018;7(1):21-27.

4. Centers for Medicare & Medicaid Services. Physician Fee Schedule Look-Up Tool. National 2021 Payment Amount by HCPCS Code. October 2021. Published 2021. Accessed February 4, 2022. https://www.cms.gov/Medicare/Medicare-Fee-for-Service-Payment/ClinicalLabFeeSched/Clinical-Laboratory-Fee-Schedule-Files

5. Centers for Medicare & Medicaid Services. Hospital Outpatient PPS Payment by HCPCS Code for CY 2021 - Q4 October File. Accessed February 4, 2022. https://www.cms.gov/Medicare/Medicare-Fee-for-Service-Payment/HospitalOutpatientPPS

6. InHealth Professional Services. *Physicians’ Fee & Coding Guide*.; 2021.

7. DC.gov. Medicaid Medical Fee Schedule September 18, 2021. Published 2021. Accessed September 23, 2021. https://www.dc-medicaid.com/dcwebportal/nonsecure/feeScheduleDownload

8. Agency for Healthcare Research and Quality Healthcare Cost and Utilization Project (HCUP). Healthcare cost and utilization project — HCUP. A federal-state-industry partnership in health data. Published 2020. Accessed September 29, 2021. https://www.hcup-us.ahrq.gov/db/nation/nis/NISIntroduction2018.pdf

9. InHealth Professional Services. 2021 Physicians’ Fee and Coding Guide (Payment Range). 2021.

10. EMD Serono. AMCP dossier for TEPMETKO® (tepotinib) in metastatic non-small cell lung cancer with MET exon14 skipping alterations. Data on file. 2022.

11. Novartis. TABRECTA (capmatinib) Prescribing Information. Published 2022. Accessed February 4, 2022. https://www.novartis.us/sites/www.novartis.us/files/tabrecta.pdf

12. US Food and Drug Administration. TEPMETKO (tepotinib) Prescribing Information. Published 2021. Accessed February 4, 2022. https://www.accessdata.fda.gov/drugsatfda_docs/label/2021/214096s000lbl.pdf

13. IBM. IBM Micromedex RED BOOK. Accessed February 4, 2022. https://www.ibm.com/uk-en/products/micromedex-red-book

14. Pfizer. XALKORI (crizotinib) Prescribing Information. Published 2022. Accessed February 4, 2022. https://www.novartis.us/sites/www.novartis.us/files/tabrecta.pdf

15. Genentech. TECENTRIQ (atezolizumab) Prescribing Information. Published 2022. Accessed February 4, 2022. https://www.gene.com/download/pdf/tecentriq_prescribing.pdf

16. Bristol Myers Squibb. OPDIVO (nivolumab) Prescribing Information. Published 2021. Accessed February 4, 2022. https://packageinserts.bms.com/pi/pi_opdivo.pdf

17. Merck Sharp & Dohme Corp., a subsidiary of Merck & Co. I. KEYTRUDA (pembrolizumab) Prescribing Information. Published 2022. Accessed February 4, 2022. https://www.merck.com/product/usa/pi_circulars/k/keytruda/keytruda_pi.pdf

18. Socinski MA, Jotte RM, Cappuzzo F, et al. Atezolizumab for first-line treatment of metastatic nonsquamous NSCLC. *N Engl J Med*. 2018;378(24):2288-2301.

19. Sandler A, Gray R, Perry MC, et al. Paclitaxel–Carboplatin Alone or with Bevacizumab for Non–Small-Cell Lung Cancer. *N Engl J Med*. 2006;355(24):2542-2550.

20. Rodríguez-Abreu D, Powell SF, Hochmair MJ, et al. Pemetrexed plus platinum with or without pembrolizumab in patients with previously untreated metastatic nonsquamous NSCLC: protocol-specified final analysis from KEYNOTE-189. *Ann Oncol*. 2021;32(7):P881-895.

21. Barlesi F, Scherpereel A, Rittmeyer A, et al. Randomized phase III trial of maintenance bevacizumab with or without pemetrexed after first-line induction with bevacizumab, cisplatin, and pemetrexed in advanced nonsquamous non-small-cell lung cancer: AVAPERL (MO22089). *J Clin Oncol*. 2013;31(24):3004-3011.

22. Shimizu T, Nakagawa Y, Asai Y, Tsujino I, Takahashi N, Gon Y. A phase II study of the combination of docetaxel and bevacizumab for previously treated non-small cell lung cancer. *J Int Med Res*. 2019;47(7):3079-3087.

23. Stefanou D, Stamatopoulou S, Sakellaropoulou A, et al. Bevacizumab, pemetrexed and carboplatin in first-linetreatment of non-small cell lung cancer patients: Focus on patients with brain metastases. *Oncol Lett*. 2016;12(6):4635-4642.

24. Sanofi-Aventis. DOCETAXEL injection, for intravenous use. Prescribing Information. Published 2021. Accessed February 4, 2022. https://products.sanofi.us/Docetaxel/Docetaxel.pdf

25. Eli Lilly. ALIMTA (pemetrexed for injection), for intravenous use. Prescribing Information. Published 2019. Accessed February 4, 2022. http://pi.lilly.com/us/alimta-pi.pdf

26. Gervais R, Robinet G, Clément-Duchêne C, et al. Pemetrexed and carboplatin, an active option in first-line treatment of elderly patients with advanced non-small cell lung cancer (NSCLC): A phase II trial. *Lung Cancer*. 2013;80(2):185-190.

27. NICE. NICE health technology evaluations: the manual (PMG36). Published online January 31, 2022. Accessed August 29, 2022. https://www.nice.org.uk/process/pmg36/resources/nice-health-technology-evaluations-the-manual-pdf-72286779244741

28. Van Hout B, Janssen MF, Feng YS, et al. Interim scoring for the EQ-5D-5L: mapping the EQ-5D-5L to EQ-5D-3L value sets. *Value Health*. 2012;15(5):708-715.

29. Shaw JW, Johnson JA, Coons SJ. US valuation of the EQ-5D health states: development and testing of the D1 valuation model. *Med Care*. 2005;43(3):203-220.

30. Institute for Clinical and Economic Review. Treatment Options for Advanced Non-Small Cell Lung Cancer: Effectiveness, Value and Value-Based Price Benchmarks. Published 2016. Accessed February 4, 2022. https://icer.org/wp-content/uploads/2020/10/MWCEPAC_NSCLC_Final_Evidence_Report_Meeting_Summary_110116.pdf

31. National Institute for Health and Care Excellence. Nivolumab for previously treated non-squamous non-small-cell lung cancer: Technology appraisal guidance [TA484]. Published 2017. Accessed February 4, 2022. https://www.nice.org.uk/guidance/ta484

32. Centers for Medicare & Medicaid Services. Medicare Q4-2020 Clinical Diagnostic Laboratory Fee Schedule. 2020.

33. Huang M, Lopes GDL, Insinga RP, et al. Cost-effectiveness of pembrolizumab versus chemotherapy as first-line treatment in PD-L1-positive advanced non-small-cell lung cancer in the USA. *Immunotherapy*. 2019;11(17):1463-1478.

34. NICE. Crizotinib for treating ROS1-positive advanced non-small-cell lung cancer. 2018. https://www.nice.org.uk/guidance/ta529

35. NICE. Osimertinib for untreated EGFR mutation-positive non-small-cell lung cancer. 2020. https://www.nice.org.uk/guidance/ta621

36. Sanders GD, Neumann PJ, Basu A, et al. Recommendations for conduct, methodological practices, and reporting of cost-effectiveness analyses: Second panel on cost-effectiveness in health and medicine. *JAMA*. 2016;316(10):1093-1103.

37. EMD Serono. FUSION: Flatiron Unmet Need Study In Clinical Outcomes among METex14 Skipping Non-Small Cell Lung Cancer. Data on file. 2020.

38. EMD Serono. Tepotinib Phase II in NSCLC harboring MET alterations. Clinical Study Report. Data on File. 2020.
